# *Verrucomicrobiota* are specialist consumers of sulfated methyl pentoses during diatom blooms

**DOI:** 10.1038/s41396-021-01105-7

**Published:** 2021-09-07

**Authors:** Luis H. Orellana, T. Ben Francis, Marcela Ferraro, Jan-Hendrik Hehemann, Bernhard M. Fuchs, Rudolf I. Amann

**Affiliations:** 1 Department of Molecular Ecology, Max Planck Institute for Marine Microbiology, Bremen, Germany; 2 Center for Marine Environmental Sciences, MARUM, University of Bremen, Bremen, Germany

**Keywords:** Water microbiology, Metagenomics, Microbial ecology

## Abstract

Marine algae annually sequester petagrams of carbon dioxide into polysaccharides, which are a central metabolic fuel for marine carbon cycling. Diatom microalgae produce sulfated polysaccharides containing methyl pentoses that are challenging to degrade for bacteria compared to other monomers, implicating these sugars as a potential carbon sink. Free-living bacteria occurring in phytoplankton blooms that specialise on consuming microalgal sugars, containing fucose and rhamnose remain unknown. Here, genomic and proteomic data indicate that small, coccoid, free-living *Verrucomicrobiota* specialise in fucose and rhamnose consumption during spring algal blooms in the North Sea. *Verrucomicrobiota* cell abundance was coupled with the algae bloom onset and accounted for up to 8% of the bacterioplankton. Glycoside hydrolases, sulfatases, and bacterial microcompartments, critical proteins for the consumption of fucosylated and sulfated polysaccharides, were actively expressed during consecutive spring bloom events. These specialised pathways were assigned to novel and discrete candidate species of the *Akkermansiaceae* and *Puniceicoccaceae* families, which we here describe as *Candidatus* Mariakkermansia forsetii and *Candidatus* Fucivorax forsetii. Moreover, our results suggest specialised metabolic pathways could determine the fate of complex polysaccharides consumed during algae blooms. Thus the sequestration of phytoplankton organic matter via methyl pentose sugars likely depend on the activity of specialised *Verrucomicrobiota* populations.

## Introduction

Polysaccharides are a diverse class of macromolecules consisting of different monomeric building blocks, linkage types and various chemical substitutions. Their structures can be linear to highly branched, creating unparalleled molecular diversity [1]. Polysaccharides are used as an intracellular energy store, cell wall material for mechanical strength and for cell-cell communication [2]. Owing to their chemical diversity these molecules may induce resource partitioning, where heterotrophic microbial taxa specialise on certain types of polysaccharides [3]. Understanding resource partitioning is important since polysaccharide-specialised microbes could dictate biomass turnover and therefore carbon storage potential of polysaccharides in the ocean.

Polysaccharide resource partitioning may be present in marine microbial communities. For instance, during spring phytoplankton blooms, peaks of net primary production are characterised by a substantial release of organic matter [4–6]. Diverse groups of marine bacteria rapidly consume labile polysaccharide types [7, 8]. The ubiquitous presence of laminarin [9] and the limited set of genes required for its degradation into glucose may render it a suitable molecule for generalist polysaccharide consumers. Other polysaccharides require more enzymes to degrade and are only accessible to specialised bacteria [8]. These polysaccharides are slowly or not fully degraded, potentially enabling either transport of carbon and other elements within sinking particles into marine sediments, or persistence within dissolved oceanic carbon sinks [8, 10]. Presence of microbes with the right enzymes likely controls the quantity and types of organic molecules that are sequestered within these two carbon pools, which contain, on average, up to 30% of polysaccharides [11, 12].

Diatoms synthesise and secrete fucose-containing sulfated polysaccharides (FCSPs) as dissolved molecules which aggregate into particles and may sequester carbon during phytoplankton blooms [8, 13]. Although these FCSP particles accumulate during diatom blooms, indicating they are not degraded by bacteria, fucosidase genes found in metagenomic samples during spring blooms could be involved in FCSP degradation [8, 14]. A recently documented macroalgae-associated *Verrucomicrobiota* isolate employs many glycoside hydrolases (GHs), sulfatases, and bacterial microcompartments (BMCs) for the degradation of FCSPs [15]. However, microbes that consume diatom FCSP, which may have different structural features compared to macroalgal FCSP, remain unknown. Recent studies have documented an abundance of fucosidase and sulfatase genes in *Verrucomicrobiota* (formerly *Verrucomicrobia*) that may play a key role in the degradation of stable polysaccharide such as FCSPs [15–21]. Similarly, *Verrucomicrobiota* metagenome-assembled genomes (MAGs) from freshwater are characterised by high content of GHs with predicted methyl pentose degradation activity (e.g. α-L-fucosidase and α-L-rhamnosidase) [19, 20]. Similar features have also been observed in MAGs recovered from sponges [22]. Intriguingly, our previous taxonomic characterisation of the surface microbial communities in Helgoland indicate that *Verrucomicrobiota* are strictly seasonal, and distinctive populations were found during the spring blooms, implying their involvement in the turnover of fucose-containing polysaccharides [23].

Here, we asked whether *Verrucomicrobiota* contribute to the catabolism of sulfated and fucose-containing polysaccharides during spring phytoplankton blooms. We combined fluorescence in situ hybridisation (FISH), metagenomic, and metaproteomic approaches to characterise *Verrucomicrobiota* populations recovered from the North Sea in 2010, 2011, 2012 and 2016. We found discrete *Verrucomicrobiota* populations carrying specialised and active pathways for the degradation of fucose and rhamnose. These findings suggest substrate partitioning between heterotrophic bacteria with respect to labile and stable polysaccharides and, moreover, a central role for *Verrucomicrobiota* in the remineralisation of complex polysaccharides.

## Materials and methods

### Sampling and sequencing

Surface seawater samples were collected from the ‘Kabeltonne’ long-term ecological research station off the North Sea island of Helgoland (54°11.3’ N, 7°54.0’ E) as described previously [14, 24]. Briefly, to remove most phytoplankton and particle-associated microorganisms, water samples were passed through 10 and 3 µm pore-size filters and cells were collected on 0.2 µm pore-size polycarbonate filters. Collected seawater for cell counting was not fractionated. The sequencing of 2010, 2011, 2012 and 2016 surface water metagenomes was performed at the DOE Joint Genome Institute (DOE-JGI) as described previously [24, 25]. All metagenomes were sequenced on a HiSeq platform (Illumina, San Diego, CA, USA) with paired-end sequencing. Trimming and processing of all raw reads was performed as previously described [24, 25].

### Short-read assembly and MAG recovery

Helgoland MAGs were obtained from previously assembled (i.e. years 2010, 2011 and 2012) Helgoland metagenomes [24, 25]. MAGs from 2016 metagenomes were previously generated using the same methodology [26]. Briefly, short-reads were de novo assembled using SPADES v3.10 [27] (meta option) and contigs longer than 2.5 kbp were binned using CONCOCT [28]. All MAGs were first filtered based on their completeness and contamination determined by checkM [29] (%[completion] − 5*[%contamination] > 50). MAGs were subsequently de-replicated using an average nucleotide identity (ANI) cut-off of 95% and alignment fraction of 65% [30] as determined by FastANI v1.1 [31]. Representative MAGs for each cluster of genomes sharing over 95% ANI were selected as the MAG with the higher quality value based on its completeness, contamination and N50 (quality = %[completion] − 5*[%contamination] + 1/2 log10(N50)) using graphs as implemented in Cytoscape v3.7.1 [32] as previously reported. A total of 440 representative MAGs were sequentially labelled (e.g. from r1 to r440; Table S1). MAGs belonging to the *Verrucomicrobiota* were selected according to their phylogenetic placement using checkM [29] and corroborated in GTDB-tk v1.0.2 [33]. Representative *Verrucomicrobiota* MAGs were also selected on their quality (see above) but MAGs belonging to the same ANI cluster were considered when 16S rRNA genes were detected (thus MAGs with higher scores but lacking 16S rRNA genes were not selected if another MAG encoded a 16S rRNA gene). An additional inspection for congruent genetic composition and coverage for all representative *Verrucomicrobiota* MAGs was performed in anvi’o v5 [34] and updated genomic sequences were updated at the European Nucleotide Archive (Study PRJEB28156). *Verrucomicrobiota* MAGs were numerically labelled but a leading letter c was used (e.g. Pun4) to differentiate from the original group that was not subjected to preferential selection of MAGs encoding 16S rRNA gene sequences and refinement. To capture a higher intra-population diversity the MAGs within the *Verrucomicrobiota* group (*n* = 182) were de-replicated at 99% ANI resulting in 26 representatives. The two additional MAGs Pun8 and Akk8 originated from the clusters Pun4 and Akk7, respectively. Thus, a total of 26 MAGs (ANI99) and 24 MAGs (ANI95) were determined depending on the ANI cut-off (Table S2). Gene coding sequences for representative MAGs were predicted using Prokka v1.14.6 [35] and taxonomic classifications were determined using GTDB-tk v1.0.2 [36] and the GTDB release r89. MAG abundances were determined as described previously [37]. Briefly, MAG abundances were determined as the quotient between the truncated average sequencing depth (TAD) of each MAG and the sequencing depth of the *rpoB* gene in each metagenome. Predicted protein sequences encoding *rpoB* genes were searched using a manually curated database previously published [38].

Previously recovered *Verrucomicrobiota* MAGs and genomes were obtained from freshwater [19–21, 39], marine water [40], and the GTDB repository (phyla *Verrucomicrobiota* and Verrucomicrobiota_A) [36]. All MAGs were quality filtered (%[completion] − 5*[%contamination] > 50) and de-replicated using FastANI and a 99% ANI as value cut-off (65% minimum genome aligned), reducing the collection from 1907 to 1024 de-replicated MAGs. Representative MAGs for each of 99% ANI groups were selected based on the highest completion and lowest contamination and visualised in Cytoscape v3.7.1 as previously reported [25]. A complete list of the 1024 genomes is available at https://github.com/lhor/Verrucomicrobiota-Helgoland

### MAG and gene phylogenies

The phylogenies for the bacterial MAG representatives from Helgoland and *Verrucomicrobiota* MAGs were constructed by first searching, extracting, and aligning a collection of 120 single-copy marker protein sequences obtained from GTDB-tk [33] and using ClustalΩ [41]. Maximum-likelihood phylogenetic estimations were determined using the collection of single-copy marker protein sequences in FastTree v2.1.10 (options -gamma -lg) [42] and visualised in the interactive Tree of Life (iTol) [43].

A *Verrucomicrobiota* collection of 1551 16S rRNA genes was obtained from SILVA [44]. Sequences encoding 16S rRNA genes in MAGs were detected and extracted using Barrnap v0.9 (https://github.com/tseemann/barrnap). An additional 25 sequences belonging to *Verrucomicrobiota* obtained from Helgoland were added (see Data availability for accession numbers). The 16S rRNA gene for the recently described *Lentimonas sp*. CC4 was also added [15]. All sequences were aligned using SINA v1.6.1 [45] and conserved gaps and ambiguously aligned regions were removed from the alignment using TrimAl v1.4.rev15 (option -gt 0.99). The generated alignment was used for a maximum-likelihood reconstruction in RAxML v8.2.12 [46] using a GTRCAT model and 1000 bootstraps.

### Gene annotation and analyses

Predicted protein sequences from each MAG were annotated using EggNOG 4.5 [47] and selected TIGRFAM [48] and PFAM [49] HMM models (See Table S3 for specific databases and HMM models used). Peptidases were searched using predicted protein sequences and the MEROPS database (downloaded April 2018; Table S4) [50] and BLASTp 2.10.0 + [51] selecting for matches with >40% identity and >50% query aligned (all MEROPs annotations were considered). Membrane transport proteins were searched using BLASTp (>40% identity and >50% query aligned) and the Transporter Classification Database (TCDB) [52]. Predicted protein sequences from MAGs encoding carbohydrate-active enzymes (CAZy) [53] were searched using BLASTp (identity >40% and query alignment >50%) and HMMER3 [54] (*E*-value < 1e−15) against the dbCAN database v07312018 [55]. Predicted CAZy protein sequences detected by both tools were used for analyses as previously suggested (Table S5) [56]. Individual GH protein sequences detected in *Verrucomicrobiota* MAGs were manually confirmed using InterProScan [57]. Orthologous groups of proteins among *Verrucomicrobiota* MAGs were identified using OrthoFinder v2.3.3 [58]. A summary of the main predicted metabolic features was produced for representative *Verrucomicrobiota* MAGs (Table S3).

### Metaproteomic analyses

Metaproteomic samples obtained during the spring bloom in 2016 were previously described [26] and reanalysed here to investigate proteins derived from *Verrucomicrobiota* populations. Briefly, protein extractions from six time points were done from planktonic microbial biomass pre-filtered through 10 µm and 3 µm size filters and finally collected on 0.2 µm pore size polycarbonate filters. All samples were processed as previously reported [26]. Mass spectra are available in the PRIDE database under accession PXD019294. Predicted sequences from nine metagenomic samples previously reported [26] and obtained during the spring bloom of 2016 were used to search the MS/MS spectra. Normalised spectral abundance factor values were determined based on the number of spectral counts obtained per protein in each sample and average values were determined from three biological replicates (not identified proteins within a replicate were considered as “0” in the calculations). Predicted proteins from *Verrucomicrobiota* MAGs were searched against the sequences of proteins detected in the metaproteome using BLASTp v2.5.0 [51] and selecting matches having an identity threshold of 99% and an alignment between the query and reference equal or greater than 50%.

### Catalysed reporter deposition fluorescence in situ hybridisation

Probes were designed to target clades containing members of the Akk7, Pun4, MB1 and MB5 clusters (Table S6). The two probes targeting Pun4 were designed to capture a broad assembly of sequences previously obtained from Helgoland (Pun4b) and to specifically target the Pun4 population derived from MAGs (Pun4s; Fig. SR1 in Supplementary Results). Cell enumeration and Catalysed Reporter Deposition Fluorescence in situ hybridisation (CARD-FISH) were performed as previously reported [37, 59].

### 16S rRNA gene oligotyping

Sample collection, processing and analyses have been described previously [23, 37]. Briefly, surface water samples were filtered in two fractions corresponding to the size ranges of 0.2–3 µm and 3–10 µm. Amplicons for both size fractions were generated using PCR amplification of the 16S rRNA gene (V4 region) and sequencing using MiSeq 2 × 250 bp chemistry at the DOE-JGI. Primers used were 515 F (5′-GTGCCAGCMGCCGCGGTAA-3′) and 806 R (5′-GGACTACHVGGGTWTCTAAT-3′). A comparison between the two size fractions at different seasons was determined with the DESeq2 package [60].

## Results

### *Verrucomicrobiota* populations are enriched in fucosidases and sulfatases

**Fig. 1:**
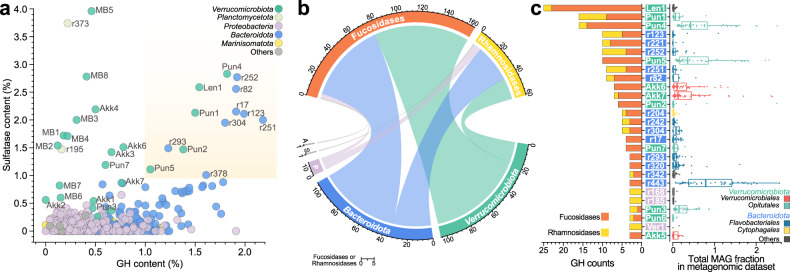
Glycoside hydrolase and sulfatase content in representative bacteria Helgoland MAGs. **a** Distribution of glycoside hydrolase (GH) and sulfatase content in 430 representative bacterial MAGs from Helgoland. Highlighted area indicates MAGs with GHs and sulfatase content equal or higher than 1% of the total predicted gene sequences. Labels starting with suffix “r” next to circles correspond to the representative number after de-replication (See the complete list in Table S1). Other labels correspond to the *Verrucomicrobiota* names according to their family affiliation: *Akkermansiaceae* (Akk), *Puniceicoccaceae* (Pun), MB11C04 (MB), and Verruco-01 (Verr). A complete taxonomic description was determined for all representative MAGs (Table S2). **b** Fucosidase and rhamnosidase sequences detected in bacterial MAGs from Helgoland. A: *Actinobacteria*, S: SAR324, P: *Proteobacteria*. Numbers around the circular layout correspond to the number of total fucosidases and rhamnosidases (top) found in different phyla (bottom). **c** Distribution of abundances for bacterial MAGs encoding a high number of fucosidases and rhamnosidases. The left side summarises the fucosidase (GH29, GH95, GH141, GH151 and GH139) and rhamnosidase (GH78, GH90 and GH106) content in MAGs. Abundance values > 0 are displayed in the right panel. Rectangles with labels in the middle are coloured according to the different phyla shown in (**a**). Box plots are coloured according to the corresponding order level for each MAG.

To investigate if verrucomicrobial enzymes play a role in the turnover of diatom polysaccharides, we first compared the composition of GHs, peptidases, and sulfatases in 430 bacterial representative MAGs obtained in Helgoland metagenomes (0.2–3 µm pore-size fraction) during 2010, 2011, 2012 and 2016 (Figs. S1a, S2 and Table S1). The fraction of predicted protein sequences annotated as GHs in bacterial MAGs had a maximum of 2.18% with a median of 0.47% of the total gene content (Fig. 1a and Fig. S1b, e). High peptidase content was characteristic of *Bacteroidota* and *Proteobacteria* but not *Verrucomicrobiota* MAGs (Fig. S1d, g). Along with *Planctomycetota* MAGs, *Verrucomicrobiota* carried the highest sulfatase content (avg = 1.44%) compared to other groups such as *Bacteroidota* (avg = 0.27%) (Fig. 1a and Fig. S1c, f).

*Verrucomicrobiota* and *Bacteroidota* MAGs accounted for ~97% (158/163) of GHs annotated as putative fucosidases mostly belonging to the GH29 and GH95 families according to the Carbohydrate Active Enzymes (CAZy) database [53]. In addition, the same bacterial phyla accounted for ~87% (53/61) of GHs annotated as putative rhamnosidases in the GH78 and GH106 families [61–63] (Fig. 1b). Twelve MAGs belonging to the *Bacteroidota* and *Verrucomicrobiota* had both high GH and sulfatase contents at >1% of their total proteins predicted in both categories (Fig. 1a). These 12 MAGs also carried ~55% (89/163) and ~43% (26/61) respectively of total GHs related to fucosidase and rhamnosidase activity detected in the 430 bacterial representative MAGs. Remarkably, 86 out of the total 163 detected fucosidases belonged to only 12 *Verrucomicrobiota* MAGs whereas 72 fucosidases detected in *Bacteroidota* were distributed in 27 MAGs. *Verrucomicrobiota* MAGs carried, on average, ~7 fucosidases per MAG compared to ~2.6 fucosidases per MAG in the *Bacteroidota*. Thus, a significantly higher fucosidase content (fucosidases/total predicted proteins) was determined for the *Verrucomicrobiota* (avg = 0.32%) compared to the *Bacteroidota* (avg = 0.12%) MAGs (Wilcoxon rank-sum test, *p* value < 0.05).

The metagenomic time-series approach allowed us to compare the relative abundances of *Verrucomicrobiota* to other populations enriched in GHs and sulfatases during the spring bloom. A total of ten out of the twelve *Verrucomicrobiota* MAGs carrying the highest GH and sulfatase content also carried the highest content of fucosidases and rhamnosidases. The *Verrucomicrobiota* MAGs of this group were, in general, more abundant compared to those from the *Bacteroidota* or others (Fig. 1c). For instance, the highest abundances were determined for *Verrucomicrobiota* MAGs Pun2, Pun4, and Pun5, individually comprising up to 2.5% of the total microbial community (Fig. 1c). In addition, an individual average abundance of ~0.6% throughout the metagenomic samples was determined for Pun4 and Pun5. However, other MAGs carrying a high number of fucosidases (e.g. r252 and r82 of the *Flavobacteriaceae* family; Fig. S3 and Supplementary Results) were less abundant and represent, on average, less than ~0.06% of the total population. Nonetheless, other *Bacteroidota* MAGs carrying lower numbers of fucosidase or rhamnosidase sequences were abundant in the metagenomic samples (e.g. r443, *Flavobacteriaceae* family; Fig. 1c), likely indicating a different niche compared to most *Verrucomicrobiota* populations, in agreement with previous reports [3].

### Three distinctive *Verrucomicrobiota* families occur during spring blooms at Helgoland

**Fig. 2:**
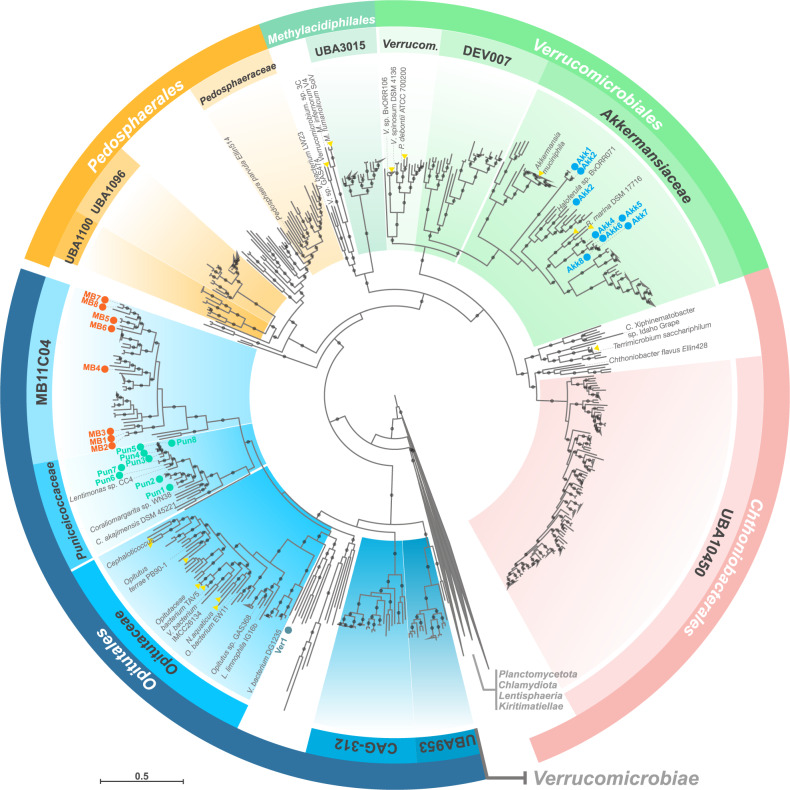
Phylogenetic reconstruction of a collection of 636 *Verrucomicrobiota* genomes including the MAGs recovered from Helgoland. The maximum-likelihood tree was based on a group of 120 conserved genes. Taxonomic classifications were determined using GTDB-tk. All orders in the external ring (coloured) belong to the *Verrucomicrobiae* class. Families containing ten or more genomes are highlighted (internal ring). Recovered MAGs from Helgoland are named according to their family affiliation and are highlighted by red circles. Branch support values between 90 and 100% are represented by dots. Names at the branches for isolates are shown in grey. Yellow triangles highlight branches for other isolates related at the genus/species level to those named. Abbreviated name in the second ring corresponds to *Verrucomicrobiaceae*. Complete names for abbreviated isolates: *Coraliomargarita akajimensis* DSM 45221, *Verrucomicrobia bacterium* IMCC26134, *Cephaloticoccus capnophilus* CV41, *Cephaloticoccus primus* CAG34, *Nibricoccus aquaticus* HZ-65, *Opitutaceae bacterium* EW11, *Lacunisphaera limnophila* IG16b, *Verrucomicrobiae bacterium* DG1235, *Rubritalea marina* DSM 17716, *Prosthecobacter debontii* ATCC 700200, *Verrucomicrobium spinosum* DSM 4136, *Verrucomicrobium* sp. BvORR106, *Methylacidiphilum infernorum* V4, *Verrucomicrobium* sp. GAS474, *Verrucomicrobia* bacterium LW23.

We sought to further characterise the *Verrucomicrobiota* MAGs and to analyse their potential for the degradation of phytoplankton-derived organic matter. A phylogenetic reconstruction using conserved single-copy protein-coding genes provided an overview of the diversity of *Verrucomicrobiota* populations at Helgoland (Fig. 2). For MAGs de-replicated at ANI ≥ 99% (Fig. S4), one MAG was classified within the *Lentisphaeria* class (Len1) and the remaining *Verrucomicrobiae* MAGs belonged to the *Akkermansiaceae* (Akk1-8), Verruco-01 (Ver1), *Puniceicoccaceae* (Pun1-8) and MB11C04 (MB1-8) families. MAGs were classified within single genera for *Puniceicoccaceae* and MB11C04 families, whereas in the *Akkermansiaceae* family three genera were detected (Table S2). MAG sizes ranged from 1.3 to 4.7 Mbp. The completion ranged from 99.3 to 70.2% and the contamination was up to 2.86%. The average G + C content for MAGs belonging to the *Puniceicoccaceae* (53%) was higher compared to the *Akkermansiaceae* (48%) and MB11C04 (43%) families (Table S2). The topology of a phylogenetic tree using recovered 16S rRNA gene sequences was congruent with the genome-based tree (Fig. S5a). A contrast between GTDB and NCBI taxonomy (Table S2) and comparisons of the *Verrucomicrobiota* MAGs to previously reported diversity were also determined (Supplementary results).

### *Akkermansiaceae*, *Puniceicoccaceae*, and MB11C04 populations are recurrent during spring phytoplankton blooms

**Fig. 3:**
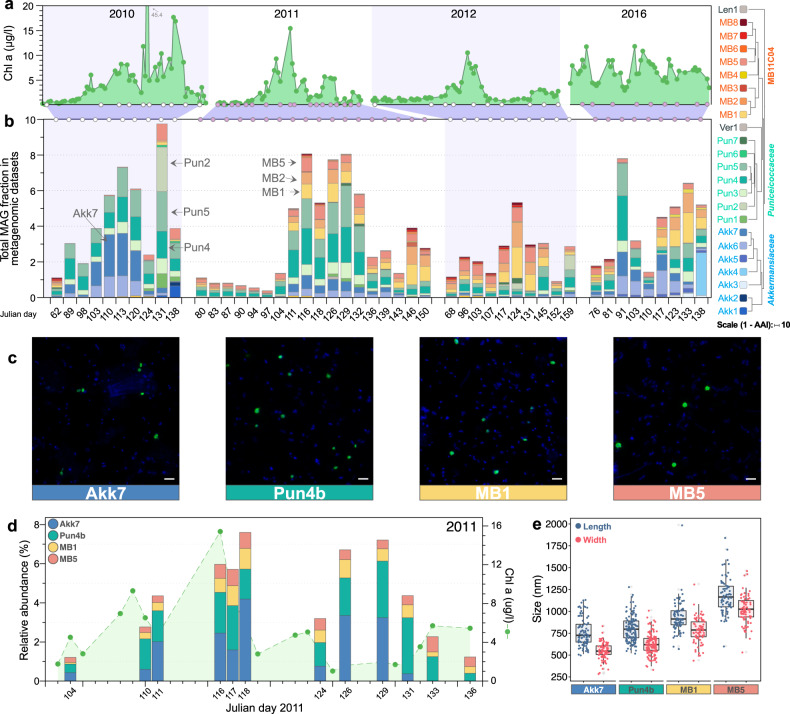
Abundance and morphology of representative *Verrucomicrobiota* MAGs in 2010, 2011, 2012 and 2016. **a** Chlorophyll a measurements for samples used for metagenomic analyses in each year. **b** Individual MAG abundance (measured as a fraction of the metagenome) for each group. Blue, green and red colour hues represent members of the *Akkermansiaceae*, *Puniceicoccaceae*, and MB11C04 families, respectively. The separation between metagenomic samples does not represent a continuous time scale on the *x*-axis (as opposed to **a**). **c** CARD-FISH results using probes to target populations Akk7 (*Akkermansiaceae*), Pun4b (*Puniceicoccaceae*), MB1 (MB11C04) and MB5 (MB11C04). Probe Pun4b was designed to target a clade comprising previously recovered sequences from Helgoland related to the Pun4 population. Scale bar represents 2 µm. **d** Abundance (relative to total cell counts) of *Verrucomicrobiota* cells. **e** Distribution of cell sizes for detected populations of *Verrucomicrobiota*.

We initially assessed the abundance and persistence of *Verrucomicrobiota* MAGs in metagenomic samples over the course of the 2010, 2011, 2012 and 2016 phytoplankton spring blooms. Similar to previously described for *Flavobacteriia*, *Gammaproteobacteria* and *Alphaproteobacteria* bacterial classes [14], recurrence was also detected for *Verrucomicrobiota* MAGs (Fig. S6a). For the most part, the increase in abundance for relatively highly abundant *Verrucomicrobiota* MAGs corresponded with the onset of the spring blooms (Fig. S6b), which was also evident when analysing the abundance of 16S rRNA genes year-round (see section below). While relative abundances determined from metagenomic samples for *Verrucomicrobiota* MAGs before the spring bloom were low (e.g. average 0.8% during 2011), members of the *Puniceicoccaceae*, MB11C04 and *Akkermansiaceae* families comprised up to 9% of the total population during the spring blooms. In particular, MAGs Akk6 and Akk7 (*Akkermansiaceae*), Pun3, Pun4 and Pun5 (*Puniceicoccaceae*), and MB5 (MB11C04) were among the most abundant species-level populations detected (Fig. 3b). While the aforementioned MAGs were persistent during the sampled periods, some members of the *Akkermansiaceae* group were only sporadically recovered (e.g. in 2010 vs. 2011). MAGs representing the populations Pun4 and Mb5 were recovered in 40 and 36 out of the 47 metagenomes analysed (Fig. 3b and Fig. S6a).

### Identification, quantification and localisation of *Verrucomicrobiota* cells

The recovery of 16S rRNA sequences from Akk7, Pun4, MB1 and MB5 MAGs allowed us to design clade-specific oligonucleotide probes and follow their distribution using CARD-FISH (Fig. S5b). Similarly to the relative abundances determined in metagenomic samples, increased cell numbers during the bloom were also noticeable when comparing cell numbers before and after the bloom onset (e.g. first time points in 2010–12). *Verrucomicrobiota* cell abundances comprised up to ~8% of the total cells (~175,000 cells/ml) at the peak of the bloom during 2011 (Fig. 3d). Cells belonging to Akk7 and Pun4b were similar in size (median length/width of ~750/550 nm), whereas cells of the MB11C04 family were larger, especially MB5 cells (median length/width of 1165/1025 nm; Fig. 3e). Clade-specific oligonucleotides identified mostly single cells that were neither accumulated in aggregates nor attached to particles (Fig. 3c).

Based on amplicon sequences, we compared relative abundances of *Verrucomicrobiota* populations in 0.2–3 µm and 3–10 µm size fractions of samples obtained during the spring blooms in 2010, 2011 and 2012 (Fig. S7). The year-round sampling during these 3 consecutive years provide a much clearer increase in abundance for *Verrucomicrobiota* populations with the onset of the spring bloom (Fig. S7). Oligotypes matching MB1 and MB5 were mostly detected in the 0.2–3 µm fraction whereas Akk7 and Akk8 populations represented a greater proportion of the *Verrucomicrobiota* populations in the 3–10 µm fractions. Oligotypes matching Pun4 were of similar frequency in both fractions. However, throughout the spring, more than 90% of the bacterial cells were found in the 0.2–3 µm fraction.

### Metabolic potential of *Verrucomicrobiota* from Helgoland

Core metabolic pathways such as glycolysis, gluconeogenesis, and TCA cycle were annotated in all *Verrucomicrobiota* MAGs, confirming a heterotrophic metabolism [19] (Table S3). MAGs MB1, MB4 and MB5 encoded a single copy of a rhodopsin, thus suggesting photoheterotrophy for some members of the MB11C04 genus, as previously observed for freshwater *Verrucomicrobiota* MAGs [19]. Interestingly, all members of the MB11C04 genus encode several components for the assembly of a bacterial flagellum, which were prominently abundant towards the end of the spring bloom in metaproteomes (Fig. S8a). In MAGs belonging to *Akkermansiaceae* and *Puniceicoccaceae* families, these genes were not detected except in MAG Pun7. Several transporter systems were detected among all MAGs, comprising up to 2.6% of the total predicted sequences, with the majority of them associated with primary active transport (P–P bond hydrolysis; Table S4). The presence of an ATP sulfurylase (*cysD/cysN*), adenylyl sulfate kinase, sulfate ABC transporters (*cysW*), and permeases (*sulP*), among others, indicated an assimilatory sulfate reduction potential for the majority of the *Verrucomicrobiota* MAGs. Similar to previously described freshwater populations [20], few genes related to the nitrogen cycle were detected in Helgoland MAGs. Among them were nitrous oxide reduction (*nosZ*, Ver1), nitrite reductase (*nirK*, Akk3), and assimilatory nitrate reduction to ammonium (*nrfA*, Len1).

### Methyl pentose metabolism

**Fig. 4:**
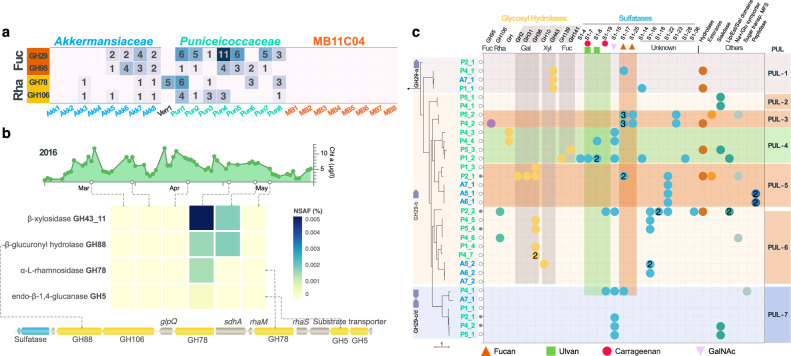
Glycoside hydrolases in *Verrucomicrobiota* MAGs. **a** Predicted number of fucosidases (Fuc) and rhamnosidases (Rha) detected in MAGs. **b** The expression of proteins related to the degradation of rhamnosidase-containing polysaccharides was detected for population representative Pun4-8 in metaproteomes towards the end of the spring bloom. The lower panel shows the genetic context of the detected proteins. β-xylosidase (GH43_11) was not part of the genetic context. **c** Summary of the phylogeny and genetic contexts for GH29 fucosidases detected in abundant and persistent *Verrucomicrobiota* MAGs. The labels in each branch correspond to the abbreviations *Akkermansiaceae* (A) and *Puniceicoccaceae* (P) MAGs. The numbers following the underscore correspond to different loci containing GH29 genes in each MAG. Each row represents the genetic context of up to ten genes upstream and downstream each GH29 gene. A filled circle next to the GH29 gene number in the tree represents a genetic context with at least ten genes upstream and downstream of a GH29 gene. Genetic contexts with <10 genes in either direction due to fragmented contigs are marked with an empty circle. Numbers inside the coloured circles represent the number of times a genetic feature was detected within the genetic context. A complete overview of all GH29 genetic contexts is also available (Fig. S9).

Transport mechanisms (proton symporters *fucP* and *rhaT*), isomerases (*fucI* and *rhamA*), mutarotases (*fucU* and *rhaM*), and kinases (*rhaB*) for the degradation of the two methyl pentoses fucose and rhamnose were prevalent in *Puniceicoccaceae* and some *Akkermansiaceae* MAGs but to a lesser extent in MB11C04 (complete list in Table S3). In addition, a high number of GHs involved in the degradation of fucose monomers were detected in *Puniceicoccaceae* and *Akkermansiaceae* MAGs (Fig. 4a and Table S5). These findings indicate that these two *Verrucomicrobiota* groups could degrade FCSPs or they enzymatically remove and use parts, such as fucose decorations, of such macromolecules for their metabolism. These polysaccharides are mainly composed of fucose and sulfate ester groups in addition to other monosaccharides such as mannose, galactose, glucose, xylose and uronic acids [64, 65]. The fucose isomerases detected in metaproteomes belonging to *Verrucomicrobiota* were prominent during the initial stages of the spring bloom in 2016, whereas xylose isomerases were detected towards the end of the bloom (Fig. S8a). The expression patterns for enzymes participating in the degradation of polysaccharides also correlated with the relative abundance of *Verrucomicrobiota* MAGs at the protein (metaproteome) and DNA (metagenome) levels (Fig. S8b).

The two most prominent GH families associated with exo-α-fucosidase activity, GH29 and GH95 [66, 67], were found in *Puniceicoccaceae* and *Akkermansiaceae* MAGs. In particular, *Puniceicoccaceae* MAGs encoded up to 11 homologs of GH29 genes per genome, compared to up to three detected homologs in *Akkermansiaceae* MAGs (Fig. 4a and Table S5). A similar number of GH95 homologs were detected among MAGs of both families. Detected GH29 sequences were divided into four orthologous groups of proteins (a, b, c/d), whereas one ortholog group contained all detected GH95 sequences (Figs. 4, S9, and S10). Syntenic arrangements for GH genes were mostly observed for closely related MAGs (i.e. high ANI), but in general the analysis of the genetic context among these ortholog groups revealed partially preserved gene arrangement which included additional orthologous groups composed of non-fucosidase GHs, sulfatases, peptidases, symporters, and carboxylesterase related genes, among others (Fig. 4c and Figs. S9, S10). Unlike in the *Bacteroidota*, no genes for SusCD machinery involved in the binding and transportation of polysaccharides were found in *Verrucomicrobiota* MAGs. However, units of co-localising fucosidases and other genes likely involved in the degradation of organic matter resembling polysaccharide utilisation loci (PULs) commonly described in *Bacteroidota* [68–70] were detected in *Verrucomicrobiota*. For instance, in five GH29-b PULs putative sugar transporters which could potentially replace SusCD in polysaccharide uptake were annotated (Figs. S9 and S10).

The co-occurrence of fucosidase genes along with other GHs and sulfatases in the same genetic contexts allowed us to predict the polysaccharide utilised by *Verrucomicrobiota* populations. In particular, the GH29 ortholog groups found in the most abundant and recurrent MAGs were separated into defined phylogenetic groups with specific sets of accessory genes (Fig. 4c). To this end, we analysed ten genes upstream and downstream of each GH29 gene used for the phylogenetic reconstruction. For each of the four ortholog GH29 groups, different PUL-like arrangements were identified based on gene similarity. The PUL-1 type mostly contains uncharacterised proteins encoding hydrolase domains (e.g. Pfam PF07859). Adjacent to the GH29a orthologues, a GH43_12 (xylosidase) was annotated, indicating the potential for the degradation of sulfated fucose/xylose polysaccharide. The majority of the remaining genetic contexts were enriched in putative sulfatases belonging to different families [71]. The GH29-c/d groups (PUL-7) likely targeting sulfated fucose were composed of two contiguous inward pointing GH29 genes, a sulfatase (S1_15), and putative sialidases (Pfam PF03629 domain). The homologs for the exo-sulfatase S1_17 found in PUL-7 and PUL-5 share more than 60% identity with biochemically characterised fucoidan sulfatase [72]. A higher gene content heterogeneity surrounding the GH29 genes was detected for the GH29-b group. Nonetheless, consistent gene content was observed within the different clades of this group. For instance, the abundant and recurrent MAGs Pun4 and Pun5, had a gene context likely targeting FCSPs (PUL-3), indicated by the presence of putative exo-sulfatases targeting fucoidans S1_17 and S1_25. These predicted protein sequences share from 40 to 62% amino acid sequence identity to homologs responding to fucoidan in the isolate *Lentimonas* sp. CC4 [15]. PUL-5 and PUL-6 were also independently enriched in sulfatases S1_22 and S1_16 respectively, both of unknown activity. Both gene contexts were also enriched in GHs with alpha- and beta-galactosidase activity (GH2, GH31 and GH36). PUL-4 and PUL-6 carried sulfatases S1_7, S1_8 and S1_19 associated with the endo- and exo- removal of sulfate in carrageenan and ulvan. However, these sulfatases share <43% identity with biochemically characterised ulvan [73] and carrageenan [74] sulfatases. PUL-4 also carried GH139 and GH141, both imparting additional α-fucosidase activity. No PULs potentially involved in the degradation of laminarin were found in the *Verrucomicrobiota* MAGs, indicating a higher specialisation for the degradation of fucose and rhamnose [20]. An overview of the genetic contexts of GH29 genes found in *Bacteroidota* MAGs was also determined (Supplementary results).

*Puniceicoccaceae* and *Akkermansiaceae* MAGs also carried GHs involved in the hydrolysis of rhamnose-containing polysaccharides. The rhamnose content of extracellular polysaccharide of diatoms can comprise up to ~40% of the dry weight content [4]. The number of potential rhamnosidases GH78 and GH106 were higher in *Puniceicoccaceae* (up to six genes) compared to single genes detected in *Akkermansiaceae* MAGs. Sequences coding for GH78 were separated into two groups of orthologs whereas GH106 sequences belonged to a single group. Rhamnosidases were surrounded by sulfatases and other GHs such as GH36 and GH2, both of which are likely galactosidases (Fig. S10). The occurrence of both GH78 and GH106 was also detected in the genetic contexts of both MAG families. Expressed GH78 of Pun8 were detected in the spring bloom in 2016, along with two other GHs in the same genetic context belonging to the GH5 (endo-β−1,4-glucanase) and GH108 (N-acetylmuramidase) families (Fig. 4b).

### Fucose and rhamnose metabolism in microcompartments

**Fig. 5:**
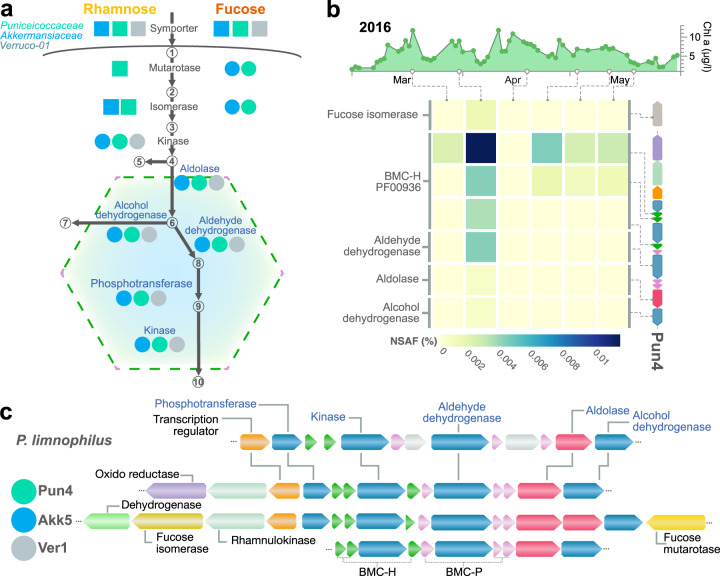
Predicted bacterial compartments in *Verrucomicrobiota* MAGs from Helgoland. **a** Proposed pathway for fucose or rhamnose in *Akkermansiaceae*, *Puniceicoccaceae* or Verruco-01 MAGs. Circles and rectangles represent the genes for different steps for the degradation pathways located in the same locus of the shell proteins (circle) or somewhere else in the genomes (rectangle). Colours indicate whether the genes encoding the different protein sequences were detected in at least one *Akkermansiaceae* (blue), *Puniceicoccaceae* (green) MAGs (complete list of detected proteins available in Table S3). Shell proteins in green and purple represent BMC-H and BMC-P components. (1) α-L-fucose, (2) β-L-fucose, (3) L-fuculose, (4) L-fuculose-1-P, (5) dihydroxyacetone phosphate (DHAP), (6) L-lactaldehyde, (7) 1,2-propanediol, (8) lactyl-CoA, (9) lactyl-phosphate, and (10) L-lactate. **b** Abundance values for proteins related to the degradation pathways of fucosidases/rhamnose and BMC components. **c** Representative BMC gene clusters for Pun4, Akk5, and Ver1 MAGs. Arrows indicate predicted genes in BMC loci and are coloured according to their predicted activity.

Protein-coding sequences necessary for the formation of catabolic BMCs were detected in *Verrucomicrobiota* MAGs belonging to the *Puniceicoccaceae* (*n* = 7/8), *Akkermansiaceae* (*n* = 4/8), and the one Ver1 family MAG. These loci are highly similar to BMCs previously experimentally characterised in *Planctomycetota*-*Verrucomicrobiota* BMCs (PV-BMC) [75] and encode the genes for the shell formation and enzymes for the aerobic degradation of fucose or rhamnose. The degradation of fucose and rhamnose in *Planctomycetes limnophilus* [75] results in the generation of lactaldehyde, a toxic metabolite. The compartmentalisation of the metabolism of fucose or rhamnose in BMCs likely prevents the cytotoxicity of the lactaldehyde molecules. The locus detected in *Verrucomicrobiota* MAGs encodes two contiguous loci containing three copies of the BMC-H (Pfam PF00936 domain) and BMC-P (Pfam PF003319 domain) shell encoding genes in a similar organisation to that previously reported [76] (Fig. 5a). These loci carry an aldolase (AraD-like) that generates lactaldehyde molecules which are processed in the BMCs (pathway details in supplementary results). The expression of proteins related to the BMC was detected in metaproteomes in 2016 (Fig. 5b, c). For instance, BMC proteins derived from MAG Pun4 related to shell proteins BMC-H (e.g. Pfam PF00936), aldehyde dehydrogenase, aldolase, and dehydrogenase were detected in metaproteomes at the abundance peak of this MAG, also coinciding with a high chlorophyll a peak in 2016. BMCs can help the metabolism of other compounds or molecules (e.g. carbon fixation in carboxysomes) [77]. Other sequences related to BMCs were detected in Helgoland MAGs but their genetic context was not linked to fucose or rhamnose degradation as in *Verrucomicrobiota* or *Planctomycetota* MAGs (Fig. S11).

Abundant *Verrucomicrobiota* carrying BMCs and PULs likely involved in the degradation of sulfated fucose or rhamnose belonged to the Akk7, Pun2, Pun4 and Pun5 populations. These four MAGs represented a maximum of 4.9% and averaged 1.6% of the microbial populations when considering the sums of their metagenomic fractions in the phytoplankton bloom of 2011. Similarly, the sum of *Akkermansiaceae* and *Puniceicoccaceae* cells determined with probes targeting Akk7 and the Pun4b clade (Fig. S5b) reached a maximum of 1.31 × 10^5^ cells/ml (April 28th, 2011) and averaged 2 × 10^4^ cells/ml during the phytoplankton blooms of 2011. Thus, methyl pentose degrading *Verrucomicrobiota* reached high relative and absolute cell numbers during spring phytoplankton blooms.

## Discussion

Our analysis predicts the partition of complex polysaccharides during algae blooms between specialised *Verrucomicrobiota* and *Bacteroidota*. Although fucosidases and rhamnosidases were identified in both phyla, a comparison solely based on gene counts is of limited value. Four lines of evidence indicate an essential role of *Verrucomicrobiota* in the degradation of FCSPs. First, *Verrucomicrobiota* populations carrying many GHs and sulfatases can be up to ten times more abundant than *Bacteroidota* populations with similar predicted polysaccharide degradation capabilities in metagenomic samples. Second, although some genetic contexts of GH29 in both groups were enriched in genes for xylanases, on average a higher diversity of co-localised GHs, specific sulfatase sub-families, and other accessory proteins in *Verrucomicrobiota* indicate a wider range for the degradation of sulfated polysaccharides. This stable assemblage of genes among different *Verrucomicrobiota* families indicates that their presence is not random and likely reflects an advantageous phenotype for the degradation of complex polysaccharides. Third, BMCs related to fucose and rhamnose degradation (e.g. PV-BMC type) were solely found in *Verrucomicrobiota* populations, indicating that methyl pentoses are a significant part of their diet. This finding indicates a niche specialisation of *Verrucomicrobiota* for methyl pentose utilisation, which is absent in *Bacteroidota*, suggesting that not only the linkage type, connectivity, and substitutions of polysaccharides but also monosaccharide composition is a niche determining trait. The data supports the hypothesis that phylum-level niche differentiation in *Verrucomicrobiota* led them to become specialised degraders of complex polysaccharides at the expense of their ability to degrade simpler polysaccharides [15]. Lastly, unlike *Bacteroidetes*, *Verrucomicrobiota* contains a lower peptidase content further supporting a strong specialisation for the degradation of fucoidan-like substrates. For instance, marine particles such as transparent exopolymers are often characterised by a high carbon/nitrogen ratio, suggesting the presence of stable polysaccharides that are difficult to degrade and lower protein content compared to algal cells or fresh algal derived organic matter [78]. Thus, the results presented here identify *Verrucomicrobiota* populations as key degraders of complex sulfated polysaccharides such as those containing fucose and rhamnose.

Previous research on pure cultures under controlled laboratory conditions has shown that BMCs are required for fucoidan degradation in *Planctomycetota* [75] and *Verrucomicrobiota* [15], preventing the accumulation of the toxic intermediate lactaldehyde during the degradation of methyl pentoses. Our bioinformatic approaches predicted that recurrent and abundant *Akkermansiaceae* and *Puniceicoccaceae* families of the *Verrucomicrobiota* populations in the North Sea carry a similar BMC locus. These results suggest a strategy for reducing toxicity when consuming polysaccharides containing fucose or rhamnose. On the other hand, members of the MBC011 family, which are also recurrent, do not carry these specialised pathways for fucose or rhamnose consumption indicating a niche that is likely independent of the utilisation of these two monosaccharides. Despite the limited number of spectra that can be associated to heterotrophic bacteria in metaproteomes during diatom blooms [26], increased levels of BMC proteins from *Verrucomicrobiota* were detected starting at the end of March in 2016. Fucose can be utilised in alternative pathways that lack lactaldehyde as an intermediate product (e.g. in *Xanthomonas campestris*) [79]. This raises the question if there is an advantage to the BMC-based degradation over the cytosol-based pathway and whether the BMC is an indicator for fucose/rhamnose-specialised bacteria. Bioenergetic studies addressing this question are missing; it may be that the BMC is especially useful when fucose and rhamnose are the major energy source and consumed in substantial quantities, which would generate lethal amounts of lactaldehyde. The accumulation of monosaccharides such as fucose and rhamnose, recently reported during the spring bloom of 2016 in Helgoland [26], can be linked to the occurrence of *Verrucomicrobiota* cells presented here. For this fucoidan-type polysaccharide, *Verrucomicrobiota* populations may regulate the rate of remineralisation during the spring phytoplankton blooms, thereby controlling a main switch of carbon sequestration. Nonetheless, the exact type of fucose-containing sulfated polysaccharide degraded by the two candidate species introduced here is still to be resolved. Recent analyses of marine *Verrucomicrobiota* isolated off the coast of Massachusetts, USA, have also determined a high capacity of these isolates to degrade FCSPs such as fucoidan. These isolates belong to the same genus of the *Puniceicoccaceae* MAGs (ANI ~80%) and share a common blueprint including many fucosidases, specific sulfatases, and BMCs. The Massachusetts isolates encode much of their genetic potential for fucoidan degradation on megaplasmids. These extrachromosomal structures were not detected in the assemblies of the metagenomic samples, likely due to technical challenges in reconstructing large plasmids from short-read metagenomic samples [80]. Alternatively, these extrachromosomal structures are not carried by *Verrucomicrobiota* populations occurring during the Spring blooms in the North Sea. Intriguingly, the *Verrucomicrobiota* MAGs assemble BMCs, fucosidases, and sulfatases on much smaller genomes. This high specialisation could also account for the low relative abundance of *Verrucomicrobiota* populations and their detected proteins. Ultimately, the results indicate that the effective consolidation of a specialised set of enzymes and a BMC results in a specific role for *Verrucomicrobiota* populations in the degradation of fucose-containing substrate during phytoplankton blooms.

### *Candidatus* Fucivorax forsetii and *Candidatus* Mariakkermansia forsetii, two specialised degraders of recalcitrant polysaccharides

As a result of this study, we have now collected information required [81] to formally describe two candidate *Verrucomicrobiota* species catalysing degradation of FCSPs with seasonal presence during phytoplankton blooms in the North Sea.

### Description of *Candidatus* Fucivorax

*Candidatus* Fucivorax (Fu.ci.vo’rax N.L. masc. n. *Fucus* genus of brown algae for which the saccharide fucose is named; L. adj. *vorax* voracious; N.L. masc. n. *Fucivorax* a voracious consumer of fucose).

Members of the genus *Ca*. Fucivorax are aerobic marine surface water bacteria. Their metabolism is predicted to be heterotrophic and specialised for the degradation of FCSPs. During the spring blooms of 2010, 2011, 2012 and 2016 in Helgoland, a total of seven species have been recovered from metagenomes, introduced here from Pun1 to Pun7. The estimated genome size and G + C content for these seven species is, on average, 2.3 Mbp and 52.8%. The genus *Ca*. Fucivorax belongs to the family *Puniceicoccaceae*, order *Opitutales*, class *Verrucomicrobiae*, and phylum *Verrucomicrobiota*. Formerly identified as genus BACL24 [33]. Type species is *Candidatus* Fucivorax forsetii and the corresponding type material is the metagenome-assembled genome Pun4.

### Description of *Candidatus* Fucivorax forsetii

*Candidatus* Fucivorax forsetii (for.se’ti.i N.L. gen. masc. n. *forsetii*, of Forseti, Scandinavian god of justice and reconciliation resident on Helgoland, from where the genome was recovered).

*Ca*. Fucivorax forsetii are observed during phytoplankton blooms in the North Sea. *Ca*. Fucivorax cells are coccoid with an average length of 803 ± 140 nm and an average width of 628 ± 114 nm. The genome annotation predicts multiple homologs of α-fucosidases, diverse sulfatase genes, and a bacterial microcompartment for the degradation of fucose-containing polysaccharides. The type material is the metagenome-assembled genome ‘20110414_Bin_47_1’(Pun4) submitted to ENA project PRJEB28156. *Ca*. Fucivorax forsetii is defined by a high-quality MAG [82] of 99.3% completion, 0% contamination, and the presence of 5S (76 bp), 16S (1,552 bp), and 23S (2,891 bp) rRNA genes and 42 tRNAs.

### Description of *Candidatus* Mariakkermansia

*Candidatus* Mariakkermansia (Ma.ri.ak.ker.man’si.a L. neut. n. *mare* sea; N.L. fem. n. *Akkermansia* genus of bacteria; N.L. fem. n. *Mariakkermansia* ocean dwelling relative of *Akkermansia*)

Members of the genus *Ca*. Mariakkermansia are predicted to be non-motile, aerobic and heterotrophic marine surface bacteria. A total of four species have been recovered from metagenomes obtained during the spring blooms of 2010, 2011, 2012 and 2016 at Helgoland introduced here from Akk4 to Akk8. The estimated genome size and G + C content for the four species are, on average, 2.25 Mbp and 50.2%. The genus *Ca*. Mariakkermansia belongs to the family *Akkermansiaceae*, order *Verrucomicrobiales*, class *Verrucomicrobiae*, and phylum *Verrucomicrobiota*. Formerly identified as genus UBA985. Type species is *Candidatus* Mariakkermansia forsetii and the corresponding type material is the metagenome-assembled genome Akk7.

### Description of *Candidatus* Mariakkermansia forsetii

*Candidatus* Mariakkermansia forsetii (for.se’ti.i N.L. gen. masc. n. *forsetii*, of Forseti, Scandinavian god of justice and reconciliation resident on Helgoland, from where the genome was recovered).

*Ca*. Mariakkermansia forsetii cells are coccoid with an average length of 764 ± 157 nm and an average width of 550 ± 91 nm. The predicted genomic potential includes α-fucosidases and a bacterial microcompartment for the degradation of fucose-containing polysaccharides. The type material for *Ca*. Mariakkermansia forsetii is the metagenome-assembled genome ‘20100303_Bin_52_1’(Akk7) submitted to ENA in the project PRJEB28156. *Ca*. Mariakkermansia forsetii is defined as a medium-quality MAG [82] due its 88.1% completion, 0% contamination, and the presence of 5 S (110 bp), 16 S (1,547 bp), and 23 S (2,838 bp) rRNA genes and 22 tRNAs.

The complete Digital Protologue Database for both proposed Candidatus species is available in Table S7. Together, all the data presented here fulfil the criteria required for the description of uncultivated prokaryotic taxa outlined by Konstantinidis et al [83].

### Outlook

*Verrucomicrobiota* populations from Helgoland share similar strategies for polysaccharide degradation to the recently described epiphyte *Lentimonas* sp. isolate [15]. However, notable differences in GH and sulfatase content along with genome size and plasmid presence propose marked differences in ecological strategies and physiological adaptations between the groups. Thus, planktonic *Verrucomicrobiota* isolates should be obtained for a comprehensive physiological characterisation to help unravel the enigmatic nature of this phylum. For instance, the determination of the substrate uptake spectra might further indicate the structural complexity and the impact of *Verrucomicrobiota* populations on the dynamics of organic matter pools during the spring blooms. In addition, a further examination into outer membrane transporters (different from the SusCD system) involved in polysaccharide transport might help us understand the mechanisms underneath the effective targeting and degradation of hard to digest polysaccharides.

## Supplementary information

Supplementary results

Supplementary figure legends

Supplementary Figure 1

Supplementary Figure 2

Supplementary Figure 3

Supplementary Figure 4

Supplementary Figure 5

Supplementary Figure 6

Supplementary Figure 7

Supplementary Figure 8

Supplementary Figure 9

Supplementary Figure 10

Supplementary Figure 11

Supplementary Table 1

Supplementary Table 2

Supplementary Table 3

Supplementary Table 4

Supplementary Table 5

Supplementary Table 6

Supplementary Table 7

## Data Availability

Metagenomes were previously deposited in NCBI (see Table S2 for BioProject accession numbers). Helgoland MAGs used in this study can be found at the European Nucleotide Archive (Study PRJEB28156). Metaproteomic data is available at PXD019294. The 16S rRNA gene sequences CAL_4_Ha_Ha_2_144 and CAL_4_HaHa_3_166 were previously deposited [84] under LR722957 and LR722956. Additional six 16S rRNA gene sequences obtained from spring blooms from Helgoland were deposited under the accession numbers LC549856, LC549863, LC549866, LC549883, LC549897, and LC549963 at the DNA Data Bank of Japan (DDBJ).

## Code availability

Code, pipelines and results used for analyses and generating figures are available on GitHub at http://github.com/lhor/Verrucomicrobiota-Helgoland

## References

[1] Laine RA (1994). A calculation of all possible oligosaccharide isomers both branched and linear yields 1.05x10(12) structures for a reducing hexasaccharide - the isomer-barrier to development of single-method saccharide sequencing or synthesis systems. Glycobiology..

[2] Varki A (2017). Biological roles of glycans. Glycobiology..

[3] Teeling H, Fuchs BM, Becher D, Klockow C, Gardebrecht A, Bennke CM (2012). Substrate-controlled succession of marine bacterioplankton populations induced by a phytoplankton bloom. Science..

[4] Myklestad SM (1995). Release of extracellular products by phytoplankton with special emphasis on polysaccharides. Sci Total Environ..

[5] Field CB, Behrenfeld MJ, Randerson JT, Falkowski P (1998). Primary production of the biosphere: integrating terrestrial and oceanic components. Science..

[6] Wetz MS, Wheeler PA (2007). Release of dissolved organic matter by coastal diatoms. Limnol Oceanogr..

[7] Reintjes G, Fuchs BM, Scharfe M, Wiltshire KH, Amann R, Arnosti C (2020). Short-term changes in polysaccharide utilization mechanisms of marine bacterioplankton during a spring phytoplankton bloom. Environ Microbiol..

[8] Vidal-Melgosa S, Sichert A, Francis TB, Bartosik D, Niggemann J, Wichels A (2021). Diatom fucan polysaccharide precipitates carbon during algal blooms. Nat Commun..

[9] Becker S, Tebben J, Coffinet S, Wiltshire K, Iversen MH, Harder T (2020). Laminarin is a major molecule in the marine carbon cycle. P Natl Acad Sci USA..

[10] Engel A, Thoms S, Riebesell U, Rochelle-Newall E, Zondervan I (2004). Polysaccharide aggregation as a potential sink of marine dissolved organic carbon. Nature..

[11] Aluwihare LI, Repeta DJ, Chen RF (1997). A major biopolymeric component to dissolved organic carbon in surface sea water. Nature..

[12] Hedges JI, Baldock JA, Gelinas Y, Lee C, Peterson M, Wakeham SG (2001). Evidence for non-selective preservation of organic matter in sinking marine particles. Nature..

[13] Meador TB, Aluwihare LI (2014). Production of dissolved organic carbon enriched in deoxy sugars representing an additional sink for biological C drawdown in the Amazon River plume. Glob Biogeochem Cycles.

[14] Teeling H, Fuchs BM, Bennke CM, Krüger K, Chafee M, Kappelmann L (2016). Recurring patterns in bacterioplankton dynamics during coastal spring algae blooms. Elife..

[15] Sichert A, Corzett CH, Schechter MS, Unfried F, Markert S, Becher D (2020). *Verrucomicrobia* use hundreds of enzymes to digest the algal polysaccharide fucoidan. Nat Microbiol.

[16] Martinez-Garcia M, Brazel DM, Swan BK, Arnosti C, Chain PS, Reitenga KG (2012). Capturing single cell genomes of active polysaccharide degraders: an unexpected contribution of *Verrucomicrobia*. PLoS ONE..

[17] Cardman Z, Arnosti C, Durbin A, Ziervogel K, Cox C, Steen AD (2014). *Verrucomicrobia* are candidates for polysaccharide-degrading bacterioplankton in an arctic fjord of Svalbard. Appl Environ Microbiol.

[18] Spring S, Bunk B, Sproer C, Schumann P, Rohde M, Tindall BJ (2016). Characterization of the first cultured representative of Verrucomicrobia subdivision 5 indicates the proposal of a novel phylum. ISME J..

[19] Cabello-Yeves PJ, Ghai R, Mehrshad M, Picazo A, Camacho A, Rodriguez-Valera F (2017). Reconstruction of diverse verrucomicrobial genomes from metagenome datasets of freshwater reservoirs. Front Microbiol.

[20] He S, Stevens SL, Chan L-K, Bertilsson S, del Rio TG, Tringe SG (2017). Ecophysiology of freshwater *Verrucomicrobia* inferred from metagenome-assembled genomes. mSphere..

[21] Tran P, Ramachandran A, Khawasik O, Beisner BE, Rautio M, Huot Y (2018). Microbial life under ice: Metagenome diversity and in situ activity of *Verrucomicrobia* in seasonally ice-covered Lakes. Environ Microbiol.

[22] Sizikov S, Burgsdorf I, Handley KM, Lahyani M, Haber M, Steindler L (2020). Characterization of sponge‐associated Verrucomicrobia: microcompartment‐based sugar utilization and enhanced toxin–antitoxin modules as features of host‐associated Opitutales. Environ Microbiol..

[23] Chafee M, Fernàndez-Guerra A, Buttigieg PL, Gerdts G, Eren AM, Teeling H (2018). Recurrent patterns of microdiversity in a temperate coastal marine environment. ISME J..

[24] Francis TB, Kruger K, Fuchs BM, Teeling H, Amann RI (2019). *Candidatus* Prosiliicoccus vernus, a spring phytoplankton bloom associated member of the *Flavobacteriaceae*. Syst Appl Microbiol..

[25] Kruger K, Chafee M, Ben Francis T, Glavina Del Rio T, Becher D, Schweder T (2019). In marine Bacteroidetes the bulk of glycan degradation during algae blooms is mediated by few clades using a restricted set of genes. ISME J..

[26] Francis TB, Bartosik D, Sura T, Sichert A, Hehemann JH, Markert S (2021). Changing expression patterns of TonB-dependent transporters suggest shifts in polysaccharide consumption over the course of a spring phytoplankton bloom. ISME.

[27] Bankevich A, Nurk S, Antipov D, Gurevich AA, Dvorkin M, Kulikov AS (2012). SPAdes: a new genome assembly algorithm and its applications to single-cell sequencing. J Comput Biol..

[28] Alneberg J, Bjarnason BS, de Bruijn I, Schirmer M, Quick J, Ijaz UZ (2014). Binning metagenomic contigs by coverage and composition. Nat Methods..

[29] Parks DH, Imelfort M, Skennerton CT, Hugenholtz P, Tyson GW (2015). CheckM: assessing the quality of microbial genomes recovered from isolates, single cells, and metagenomes. Genome Res..

[30] Varghese NJ, Mukherjee S, Ivanova N, Konstantinidis KT, Mavrommatis K, Kyrpides NC (2015). Microbial species delineation using whole genome sequences. Nucleic Acids Res..

[31] Jain C, Rodriguez RL, Phillippy AM, Konstantinidis KT, Aluru S (2018). High throughput ANI analysis of 90K prokaryotic genomes reveals clear species boundaries. Nat Commun..

[32] Smoot ME, Ono K, Ruscheinski J, Wang P-L, Ideker T (2010). Cytoscape 2.8: new features for data integration and network visualization. Bioinformatics..

[33] Chaumeil PA, Mussig AJ, Hugenholtz P, Parks DH (2019). GTDB-Tk: a toolkit to classify genomes with the Genome Taxonomy Database. Bioinformatics..

[34] Eren AM, Esen OC, Quince C, Vineis JH, Morrison HG, Sogin ML (2015). Anvi’o: an advanced analysis and visualization platform for ‘omics data. PeerJ..

[35] Seemann T (2014). Prokka: rapid prokaryotic genome annotation. Bioinformatics..

[36] Parks DH, Chuvochina M, Waite DW, Rinke C, Skarshewski A, Chaumeil PA (2018). A standardized bacterial taxonomy based on genome phylogeny substantially revises the tree of life. Nat Biotechnol.

[37] Orellana LH, Ben Francis T, Kruger K, Teeling H, Muller MC, Fuchs BM (2019). Niche differentiation among annually recurrent coastal Marine Group II Euryarchaeota. ISME J.

[38] Orellana LH, Rodriguez RL, Konstantinidis KT (2017). ROCker: accurate detection and quantification of target genes in short-read metagenomic data sets by modeling sliding-window bitscores. Nucleic Acids Res..

[39] Rodriguez RL, Tsementzi D, Luo C, Konstantinidis KT (2020). Iterative subtractive binning of freshwater chronoseries metagenomes identifies over 400 novel species and their ecologic preferences. Environ Microbiol.

[40] Delmont TO, Quince C, Shaiber A, Esen OC, Lee ST, Rappe MS (2018). Nitrogen-fixing populations of *Planctomycetes* and *Proteobacteria* are abundant in surface ocean metagenomes. Nat Microbiol..

[41] Sievers F, Higgins DG (2018). Clustal Omega for making accurate alignments of many protein sequences. Protein Sci.

[42] Price MN, Dehal PS, Arkin AP (2010). FastTree 2-approximately maximum-likelihood trees for large alignments. PLoS ONE.

[43] Letunic I, Bork P (2016). Interactive tree of life (iTOL) v3: an online tool for the display and annotation of phylogenetic and other trees. Nucleic Acids Res..

[44] Quast C, Pruesse E, Yilmaz P, Gerken J, Schweer T, Yarza P (2013). The SILVA ribosomal RNA gene database project: improved data processing and web-based tools. Nucleic Acids Res..

[45] Pruesse E, Peplies J, Glockner FO (2012). SINA: accurate high-throughput multiple sequence alignment of ribosomal RNA genes. Bioinformatics..

[46] Stamatakis A (2014). RAxML version 8: a tool for phylogenetic analysis and post-analysis of large phylogenies. Bioinformatics.

[47] Huerta-Cepas J, Szklarczyk D, Forslund K, Cook H, Heller D, Walter MC (2016). eggNOG 4.5: a hierarchical orthology framework with improved functional annotations for eukaryotic, prokaryotic and viral sequences. Nucleic Acids Res..

[48] Selengut JD, Haft DH, Davidsen T, Ganapathy A, Gwinn-Giglio M, Nelson WC (2007). TIGRFAMs and Genome Properties: tools for the assignment of molecular function and biological process in prokaryotic genomes. Nucleic Acids Res..

[49] El-Gebali S, Mistry J, Bateman A, Eddy SR, Luciani A, Potter SC (2019). The Pfam protein families database in 2019. Nucleic Acids Res..

[50] Rawlings ND, Barrett AJ, Thomas PD, Huang X, Bateman A, Finn RD (2018). The MEROPS database of proteolytic enzymes, their substrates and inhibitors in 2017 and a comparison with peptidases in the PANTHER database. Nucleic Acids Res..

[51] Camacho C, Coulouris G, Avagyan V, Ma N, Papadopoulos J, Bealer K (2009). BLAST+: architecture and applications. BMC Bioinforma.

[52] Saier MH, Reddy VS, Tsu BV, Ahmed MS, Li C, Moreno-Hagelsieb G (2016). The Transporter Classification Database (TCDB): recent advances. Nucleic Acids Res..

[53] Lombard V, Golaconda Ramulu H, Drula E, Coutinho PM, Henrissat B (2014). The carbohydrate-active enzymes database (CAZy) in 2013. Nucleic Acids Res..

[54] Eddy SR (2011). Accelerated Profile HMM Searches. PLoS Comput Biol.

[55] Yin Y, Mao X, Yang J, Chen X, Mao F, Xu Y (2012). dbCAN: a web resource for automated carbohydrate-active enzyme annotation. Nucleic Acids Res..

[56] Zhang H, Yohe T, Huang L, Entwistle S, Wu P, Yang Z (2018). dbCAN2: a meta server for automated carbohydrate-active enzyme annotation. Nucleic Acids Res..

[57] Jones P, Binns D, Chang HY, Fraser M, Li W, McAnulla C (2014). InterProScan 5: genome-scale protein function classification. Bioinformatics..

[58] Emms DM, Kelly S (2015). OrthoFinder: solving fundamental biases in whole genome comparisons dramatically improves orthogroup inference accuracy. Genome Biol.

[59] Thiele S, Fuchs B, Amann R. Identification of microorganisms using the ribosomal RNA approach and fluorescence in situ hybridization. In: Wilderer PA, editor. Treatise on Water Science. Elsevier Science; Oxford, United Kingdom; 2011. p. 171–89.

[60] Love MI, Huber W, Anders S (2014). Moderated estimation of fold change and dispersion for RNA-seq data with DESeq2. Genome Biol..

[61] Zverlov VV, Hertel C, Bronnenmeier K, Hroch A, Kellermann J, Schwarz WH (2000). The thermostable alpha-L-rhamnosidase RamA of *Clostridium stercorarium*: biochemical characterization and primary structure of a bacterial alpha-L-rhamnoside hydrolase, a new type of inverting glycoside hydrolase. Mol Microbiol.

[62] Miyata T, Kashige N, Satho T, Yamaguchi T, Aso Y, Miake F (2005). Cloning, sequence analysis, and expression of the gene encoding *Sphingomonas paucimobilis* FP2001 alpha-L -rhamnosidase. Curr Microbiol..

[63] Ndeh D, Rogowski A, Cartmell A, Luis AS, Basle A, Gray J (2017). Complex pectin metabolism by gut bacteria reveals novel catalytic functions. Nature..

[64] Li B, Lu F, Wei X, Zhao R (2008). Fucoidan: structure and bioactivity. Molecules..

[65] Ale MT, Mikkelsen JD, Meyer AS (2011). Important determinants for fucoidan bioactivity: a critical review of structure-function relations and extraction methods for fucose-containing sulfated polysaccharides from brown seaweeds. Mar Drugs..

[66] Katayama T, Sakuma A, Kimura T, Makimura Y, Hiratake J, Sakata K (2004). Molecular cloning and characterization of *Bifidobacterium bifidum* 1,2-alpha-L-fucosidase (AfcA), a novel inverting glycosidase (glycoside hydrolase family 95). J Bacteriol..

[67] Nagae M, Tsuchiya A, Katayama T, Yamamoto K, Wakatsuki S, Kato R (2007). Structural basis of the catalytic reaction mechanism of novel 1,2-alpha-L-fucosidase from *Bifidobacterium bifidum*. J Biol Chem..

[68] Anderson KL, Salyers AA (1989). Biochemical evidence that starch breakdown by *Bacteroides thetaiotaomicron* involves outer membrane starch-binding sites and periplasmic starch-degrading enzymes. J Bacteriol..

[69] Bjursell MK, Martens EC, Gordon JI (2006). Functional genomic and metabolic studies of the adaptations of a prominent adult human gut symbiont, *Bacteroides thetaiotaomicron*, to the suckling period. J Biol Chem.

[70] Grondin JM, Tamura K, Dejean G, Abbott DW, Brumer H (2017). Polysaccharide Utilization Loci: fueling microbial communities. J Bacteriol.

[71] Barbeyron T, Brillet-Gueguen L, Carre W, Carriere C, Caron C, Czjzek M (2016). Matching the diversity of sulfated biomolecules: Creation of a classification database for sulfatases reflecting their substrate specificity. PLoS ONE.

[72] Silchenko AS, Rasin AB, Zueva AO, Kusaykin MI, Zvyagintseva TN, Kalinovsky AI (2018). Fucoidan sulfatases from marine bacterium *Wenyingzhuangia fucanilytica* CZ1127(T). Biomolecules..

[73] Reisky L, Prechoux A, Zuhlke MK, Baumgen M, Robb CS, Gerlach N (2019). A marine bacterial enzymatic cascade degrades the algal polysaccharide ulvan. Nat Chem Biol..

[74] Hettle AG, Vickers C, Robb CS, Liu F, Withers SG, Hehemann JH (2018). The molecular basis of polysaccharide sulfatase activity and a nomenclature for catalytic subsites in this class of enzyme. Structure..

[75] Erbilgin O, McDonald KL, Kerfeld CA (2014). Characterization of a planctomycetal organelle: a novel bacterial microcompartment for the aerobic degradation of plant saccharides. Appl Environ Microbiol..

[76] Axen SD, Erbilgin O, Kerfeld CA (2014). A taxonomy of bacterial microcompartment loci constructed by a novel scoring method. PLoS Comput Biol..

[77] Sutter M, Melnicki MR, Schulz F, Woyke T, Kerfeld CA (2021). A catalog of the diversity and ubiquity of bacterial microcompartments. Nat Commun..

[78] Engel A, Goldthwait S, Passow U, Alldredge A (2002). Temporal decoupling of carbon and nitrogen dynamics in a mesocosm diatom bloom. Limnol Oceanogr..

[79] Yew WS, Fedorov AA, Fedorov EV, Rakus JF, Pierce RW, Almo SC (2006). Evolution of enzymatic activities in the enolase superfamily: L-fuconate dehydratase from Xanthomonas campestris. Biochemistry..

[80] Arredondo-Alonso S, Willems RJ, van Schaik W, Schurch AC (2017). On the (im)possibility of reconstructing plasmids from whole-genome short-read sequencing data. Micro Genom..

[81] Murray AE, Freudenstein J, Gribaldo S, Hatzenpichler R, Hugenholtz P, Kampfer P (2020). Roadmap for naming uncultivated Archaea and Bacteria. Nat Microbiol..

[82] Bowers RM, Kyrpides NC, Stepanauskas R, Harmon-Smith M, Doud D, Reddy TBK (2017). Minimum information about a single amplified genome (MISAG) and a metagenome-assembled genome (MIMAG) of bacteria and archaea. Nat Biotechnol..

[83] Konstantinidis KT, Rossello-Mora R, Amann R (2017). Uncultivated microbes in need of their own taxonomy. ISME J..

[84] Alejandre-Colomo C, Harder J, Fuchs BM, Rossello-Mora R, Amann R (2020). High-throughput cultivation of heterotrophic bacteria during a spring phytoplankton bloom in the North Sea. Syst Appl Microbiol.

