# Supplementary material for: Verrucomicrobiota are specialist consumers of sulfated methyl pentoses during diatom blooms
Source: ISME J. 2021 Sep 7;16(3):630–41. doi: 10.1038/s41396-021-01105-7 (PMC8857213; doi:10.1038/s41396-021-01105-7)
Supplement: Supplementary file 1 — Supplementary results [file 41396_2021_1105_MOESM1_ESM.docx]

**Supplementary Results**

**FISH**

We designed probes to specifically target Pun4 populations (Pun4s) and a broader clade composed of previously recovered sequences from Helgoland related to the Pun4 population (Pun4b; Fig. S5b). Visualised Pun4 cells using Pun4b and Pun4s followed a similar abundance pattern, but higher cell counts were determined using the former probe as expected (Fig. SR1).

**Fig. SR1.** Cell count comparison between probes Pun4b and Pun4s using CARD-FISH.

***Verruc***
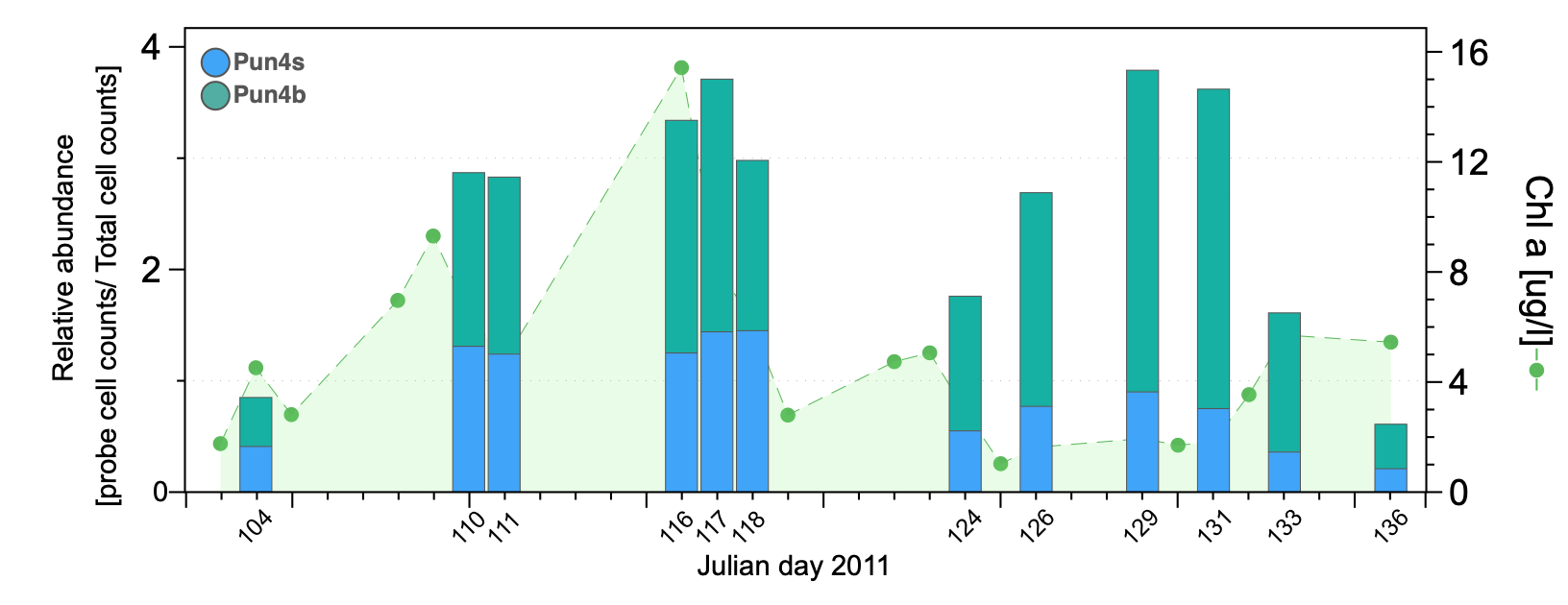
***omicrobiota* populations from Helgoland compared to previously reported genomes and MAGs.**

A comparison to 636 previously recovered *Verrucomicrobiota* isolates and MAGs further highlights the diversity of Helgoland MAGs (Fig. 2). Most recovered genomes from Helgoland shared average amino acid identity (AAI) values of ~68%-80% compared with previously recovered MAGs from marine samples, indicating their similarity at the genus level. For instance, within the *Akkermansiaceae* family (Akk4-8), most MAGs belonged to the UBA985 genus, contained in a subclade composed of genomes obtained from marine samples of the TARA and Ocean Sampling Day Consortia. MAG Akk4 shares 98% and 99.8% ANI with the MAGs UBA7329 and UBA8040 obtained from these two sampling projects, respectively. MAGs MB1, MB2, and MB3 of the MB11C04 family shared ~74%-79% AAI with MAG ARS76 recovered in the Indian Ocean of the TARA expedition. An independent clade containing the MB4 MAG shared 71.1% AAI with UBA1276 obtained in Mediterranean sea surface waters. A clade consisting of MAGs MB5-8 shared ~68%-77% AAI with MAGs obtained from the Indian Ocean (IN48 and TARA_ION_00036) and North East Atlantic (TARA_ANE_00064). MAGs of the *Puniceicoccaceae* family were located in a clade corresponding to the BACL24 genus. The 25 genomes in the BACL24 clade, but excluding MAG UBA1513 that was obtained from a sediment sample, were obtained from marine water samples. Among them, a recently described fucose-degrading isolate *Lentimonas* 1 shared 83.3% AAI (SD 18.13%, 1,693 proteins) with Pun6, the closest relative MAG from Helgoland. However, this *Lentimonas* isolate has a chromosome larger than the average member of the *Puniceicoccaceae* family (3.9 Mbp vs ~2.3 Mbp). Other closer relatives, such as UBA5964, collected from the surface of the Baltic Sea, shared an ANI of 89.2% with Pun5. Relatives of Pun1 were NP23 (TARA Ocean) and *Coraliomargarita* sp. WN38 with 75% and 68.2% AAI. In contrast, other Helgoland MAGs were closely related to those obtained from freshwater environments. For instance, MAG Akk3 of the *Akkermansiaceae* family is contained within a clade mostly composed of genomes from freshwater environments. The closest relative is the ME12612 MAG sharing 79.4% ANI, obtained from Lake Mendota, a drainage type eutrophic lake located in Wisconsin, USA 2. Other MAGs such as Ver1 (*Opitutales*) and Len1 (*Lentisphaeria*) were distantly related to MAGs in the reference set and shared less than 48% AAI with their closest relatives, indicating these genomes have a somewhat higher degree of novelty. However, a recently recovered MAG from fluids derived from deep terrestrial subsurface shale following hydraulic fracturing (West Virginia, USA)3 shares 50.1% AAI with Ver1. The phylogenetic analysis also revealed that the populations recovered in the MAGs Akk1, Akk2, Akk7, Mb5, Pun4, Pun7, Pun1, and Len1 were already present in the GTDB 4 using the same metagenomes analysed here, and which share >99% ANI. Nonetheless, these MAGs are not identical since they were obtained using different assembly and binning approaches, but they are likely to represent the same populations.

**PULs in *Bacteroidota* carrying high number of fucosidases and sulfatases**

Different genetic contexts surrounding GH29 genes were observed for *Bacteroidota* MAGs with high content of GHs and sulfatases (Fig. S3d). Overall, most differences to *Verrucomicrobiota* were in sulfatase subfamilies and GH content. Also, the presence of a SusCD system surrounding 8/23 genetic contexts carrying a GH29 gene was a clear difference to *Verrucomicrobiota* MAGs. The presence of GHs likely involved in the degradation of xylans were also detected within GH29 contexts, similar to PUL-1 in *Verrucomicrobiota* MAGs. However, a different combination of sulfatases was observed between the two bacterial phyla. *Bacteroidota* MAGs r378, r17, r82, r123, and r304 carrying high content of GHs and sulfatases also had the potential for the degradation of abundant polysaccharides such as laminarin (e.g., PULs carrying GH16 and GH3).

**Fucose utilisation and BMCs**

The results presented here indicate that fucose and rhamnose degradation is likely carried out in *Puniceicoccaceae,* *Akkermansiaceae,* and Verruco-01 MAGs using the phosphorylated pathway (5) in combination with the detoxification of lactaldehyde inside the BMCs. Despite most MAGs carrying the genes for the cytosolic reactions, only a rhamnose kinase (and not fucose kinase) was detected, also observed in sponge-associated *Verrucomicrobiota* MAGs (6). As a result of the aldolase activity, lactaldehyde and dihydroxyacetone phosphate are produced. The former compound is processed inside the BMC and it is the substrate for the aldehyde dehydrogenase that produces lactyl-CoA and NADH. The NADH can be regenerated by the activity of the alcohol dehydrogenase, producing the alcohol molecule 1,2-propanediol. The CoA moiety of the previously generated lactyl-CoA is replaced with a phosphate by a phosphotransacetylase. The phosphorylated lactate can participate in substrate-level phosphorylation. The generated lactate can now be converted to pyruvate by the lactate dehydrogenase activity also present in the same loci (Fig. 5c)

# References

1 Sichert, A. *et al.* Verrucomicrobia use hundreds of enzymes to digest the algal polysaccharide fucoidan. *Nat Microbiol*, (2020).

2 He, S. *et al.* Ecophysiology of Freshwater Verrucomicrobia Inferred from Metagenome-Assembled Genomes. *mSphere* **2**, e00277-00217, (2017).

3 Nixon, S. L. *et al.* Genome-Resolved Metagenomics Extends the Environmental Distribution of the Verrucomicrobia Phylum to the Deep Terrestrial Subsurface. *mSphere* **4**, (2019).

4 Chaumeil, P. A., Mussig, A. J., Hugenholtz, P. & Parks, D. H. GTDB-Tk: a toolkit to classify genomes with the Genome Taxonomy Database. *Bioinformatics*, (2019).

5 Erbilgin, O., McDonald, K. L. & Kerfeld, C. A. Characterization of a planctomycetal organelle: a novel bacterial microcompartment for the aerobic degradation of plant saccharides. *Appl Environ Microbiol* **80**, 2193-2205, (2014).

6 Sizikov, S. *et al.* Characterisation of sponge‐associated Verrucomicrobia: microcompartment‐based sugar utilisation and enhanced toxin–antitoxin modules as features of host‐associated Opitutales. *Environ Microbiol* **22**, 4669-4688, (2020).
