# Supplementary material for: Verrucomicrobiota are specialist consumers of sulfated methyl pentoses during diatom blooms
Source: ISME J. 2021 Sep 7;16(3):630–41. doi: 10.1038/s41396-021-01105-7 (PMC8857213; doi:10.1038/s41396-021-01105-7)
Supplement: Supplementary file 2 — Supplementary figure legends [file 41396_2021_1105_MOESM2_ESM.docx]

**Supplementary Figures**

**Figure S1.** Glycoside hydrolase, sulfatase, and peptidase content in 430 bacterial MAG representatives. **a**, Phylogenetic reconstruction of 430 bacterial MAGs using 120 conserved marker genes as implemented in GTDB-tk. Panels **b, c**, and **d** summarise glycoside hydrolase, sulfatase, and protease content in bacterial MAGs. Panels e, f, and g summarise the gene counts for the same previous genetic features. Each dot represents one MAG coloured according to their phylogenetic affiliation at the phylum level.

**Figure S2**. Abundance for all representative MAGs from Helgoland in metagenomic samples obtained during the spring blooms in 2010, 2011, 2012, and 2016. The x-axis in both figures represents the Julian days for each year.

**Figure S3**. Abundance and genetic contexts for *Bacteroidota* MAGs (r17, r82, r123, r252, r293, r304, r378) encoding a high fraction of GHs and sulfatases. **a** Chlorophyll a levels for spring blooms in four years. **b** and **c** Abundances of *Bacteroidota* and *Verrucomicrobiota* MAGs encoding high fractions of GHs and sulfatases. **d,** Genetic contexts for GH29 genes found in *Bacteroidota* MAGs encoding a high fraction of GHs and sulfatases. Unfilled symbols represents a SusCD system more than 10 genes apart from the GH29 gene.

**Figure S4**. ANI and AAI values (%) between all *Verrucomicrobiota* MAGs recovered from Helgoland. Shaded rectangles highlight values ≥ 90%.

**Figure S5**. **a**, Phylogenetic reconstruction using 1,581 *Verrucomicrobiae* 16S rRNA gene sequences including those recovered from Helgoland MAGs. **b**, Comparison of probes targeting Pun4 populations. The dashed line rectangle highlights a clade targeted by the Pun4b probe consisting of closely related sequences mostly retrieved from Helgoland (Supplementary Results). The target of the Pun4s probe (i.e., the 16S rRNA gene sequence of the Pun4 MAG) is highlighted in bold.

**Figure S6**. **a,** Recovery of *Verrucomicrobiota* MAGs in Helgoland metagenomic samples. Coloured cells indicate the recovery of a specific MAG in metagenomic samples. **b,** Sample correlation coefficients (*r*^2^) between MAG abundances and chlorophyll a values. Linear regression between MAG abundances and chlorophyll measurements was determined for each MAG abundance for values above 0.01% and normality of the data sufficed (*p* value <0.05 in the Shapiro-Wilk test). Missing *r^2^* values correspond to MAG abundance values significantly different from a normal distribution (*p* value >0.05, Shapiro-Wilk test). The labels used for the MAGs correspond to the abbreviations *Akkermansiaceae* (A), *Puniceicoccaceae* (P), MB11C04, Verruco-1 (V), and *Lentisphaeria* (L).

**Figure S7**. Distribution of *Verrucomicrobiota* oligotypes during 2010, 2011, and 2012 at Helgoland. Oligotypes were selected based on the relative abundance of at least 0.1% in at least one sample. **a**, Chlorophyll a levels and relative abundance for *Verrucomicrobiota* oligotypes in 0.2-3 μm and 3-10 μm size fractions. Oligotype sequences with perfect matches to 16S rRNA genes recovered from MAGs are also shown (Akk7, Akk8, Pun4, MB4, MB5). **b**, Log2-fold values between 0.2 - 3 μm (top) and 3-10 μm (bottom) size fractions for oligotypes that matched 16S rRNA recovered from MAGs.

**Figure S8**. Proteomic profiles for predicted functions in *Verrucomicrobiota* MAGs. **a**, Expression values for proteins related to degradation of fucose, xylose, and flagella in MAGs. **b**, Abundance values for all detected proteins related to *Verrucomicrobiota* MAGs in NSAF (%). Lower panel shows the abundance values for the same MAGs from the same samples as a fraction of the metagenome. Both figures show the results for metaproteomes obtained in 03/17, 03/31, 04/19, 05/03, 05/12, and 05/17.

**Figure S9.** Genomic contexts for fucosidase genes detected in representative *Verrucomicrobiota* MAGs. Orthologue groups of gene labels indicate the GH type (e.g. GH29 and GH95) and are followed by a letter to separate different groups. The four orthologous groups of GH29 protein sequences detected in *Verrucomicrobiota* MAGs were coloured in purple and labeled as GH-a, GH-b, GH-c, and GH-d. The labels used for fucosidase genes in each genetic context correspond to the abbreviations *Akkermansiaceae* (A) and *Puniceicoccaceae* (P) MAGs. These representations correspond to the complete genetic context summarised in the main article (Fig 4).

**Figure S10.** Genomic contexts for rhamnosidase genes detected in representative *Verrucomicrobiota* MAGs. The two orthologue groups for GH78 (a,b) and GH106 (a,b) are shown.

**Figure S11.** BMC proteins found in non-*Verrucomicrobiota* MAGs from Helgoland. **a**, Summary of genetic features related to BMCs and other related proteins found in Helgoland MAGs. Numbers separated by “|” indicate that proteins were found in different contigs. The value “Contiguous?” denotes whether the predicted proteins are part of the same genetic context. **b**, Genetic context of BMC proteins found in r342. A similar organisation is also observed in r198.

**Supplementary Tables**

**Table S1**. Representative MAGs from 2010, 2011, 2012, and 2016 metagenomes de-replicated at 95% ANI.

**Table S2.** Extended information for *Verrucomicrobiota* MAGs from Helgoland. NCBI taxonomy was determined using 16S rRNA gene sequences carried by MAGs Akk7, Pun4, MB1, MB4, and MB5 (marked with an asterisk). Taxonomy for Ver1 was determined based on 50.1% AAI shared with *Ca.* Marcellius.

**Table S3.** Annotation of main processes detected in representative *Verrucomicrobiota* MAGs from Helgoland.

**Table S4.** Classification of membrane transport proteins using the Transporter Classification Database (TCDB) for *Verrucomicrobiota* MAGs.

**Table S5**. Summary of CAZymes detected in representative *Verrucomicrobiota* from Helgoland.

**Table S6**. Probes used for visualisation of *Verrucomicrobiota* populations from Helgoland.

**Table S7**. Digital Protologue for *Verrucomicrobiota* populations Akk7 and Pun4 from Helgoland.
