# Supplementary material for: Verrucomicrobiota are specialist consumers of sulfated methyl pentoses during diatom blooms
Source: ISME J. 2021 Sep 7;16(3):630–41. doi: 10.1038/s41396-021-01105-7 (PMC8857213; doi:10.1038/s41396-021-01105-7)
Supplement: Supplementary file 6 — Supplementary Figure 4 [file 41396_2021_1105_MOESM6_ESM.pdf]

AAI

a

	L1	Pl1	Pl2	V1	A1	A2	A7	A8	A6	A5	A4	A3	M6	M5	M8	M7	M4	M2	M1	M3	P7	P2	P6	P8	P5	P4	P3	P1
L1	100	38	37	38	38	38	40	40	40	38	39	39	37	39	39	36	39	39	39	39	39	39	39	39	40	41	39	40
Pl1	38	100	36	35	37	36	36	36	36	36	37	37	37	39	38	36	37	37	37	37	36	36	36	36	36	37	36	36
Pl2	37	36	100	35	35	35	37	36	37	37	37	37	36	38	37	36	36	36	37	37	36	36	35	36	36	37	35	36
V1	38	35	35	100	39	39	40	40	40	40	40	40	43	43	43	42	43	43	43	44	49	48	46	48	49	49	47	49
A1	38	37	35	39	100	85	48	47	48	48	48	48	39	41	40	39	40	40	40	41	41	41	40	41	41	41	40	41
A2	38	36	35	39	85	100	47	47	48	47	47	47	39	40	39	38	40	40	39	40	40	41	39	40	40	41	39	40
A7	40	36	37	40	48	47	100	94	92	81	67	53	40	42	41	39	41	41	41	41	43	42	41	42	43	44	41	43
A8	40	36	36	40	47	47	94	100	90	81	67	52	39	41	40	39	40	41	41	41	42	42	41	42	43	43	40	42
A6	40	36	37	40	48	48	92	90	100	82	68	53	40	42	41	39	41	41	41	41	43	43	41	42	43	44	41	43
A5	38	36	37	40	48	47	81	81	82	100	68	53	40	41	40	39	41	40	41	41	42	42	40	42	42	42	41	42
A4	39	37	37	40	48	47	67	67	68	68	100	53	40	42	41	40	41	41	41	41	42	42	41	42	42	43	41	42
A3	39	37	37	40	48	47	53	52	53	53	53	100	41	42	41	40	41	41	41	42	42	43	42	43	42	43	42	42
M6	37	37	36	43	39	39	40	39	40	40	40	41	100	75	71	69	63	61	61	61	46	47	44	47	47	47	46	46
M5	39	39	38	43	41	40	42	41	42	41	42	42	75	100	74	71	65	62	62	62	47	47	45	47	47	48	46	47
M8	39	38	37	43	40	39	41	40	41	40	41	41	71	74	100	82	62	60	60	60	46	46	44	46	46	47	45	46
M7	36	36	36	42	39	38	39	39	39	39	40	40	69	71	82	100	60	58	58	59	45	45	43	45	45	46	44	45
M4	39	37	36	43	40	40	41	40	41	41	41	41	63	65	62	60	100	62	62	62	47	47	44	47	47	47	46	47
M2	39	37	36	43	40	40	41	41	41	40	41	41	61	62	60	58	62	100	92	75	47	47	45	46	46	47	46	47
M1	39	37	37	43	40	39	41	41	41	41	41	41	61	62	60	58	62	92	100	75	47	47	45	47	47	48	46	47
M3	39	37	37	44	41	40	41	41	41	41	41	42	61	62	60	59	62	75	75	100	48	48	45	47	48	48	46	48
P7	39	36	36	49	41	40	43	42	43	42	42	42	46	47	46	45	47	47	47	48	100	75	74	77	77	77	77	69
P2	39	36	36	48	41	41	42	42	43	42	42	43	47	47	46	45	47	47	47	48	75	100	74	78	79	78	77	68
P6	39	36	35	46	40	39	41	41	41	40	41	42	44	45	44	43	44	45	45	45	74	74	100	77	78	78	77	66
P8	39	36	36	48	41	40	42	42	42	42	42	43	47	47	46	45	47	46	47	47	77	78	77	100	97	92	89	68
P5	40	36	36	49	41	40	43	43	43	42	42	42	47	47	46	45	47	46	47	48	77	79	78	97	100	94	89	70
P4	41	37	37	49	41	41	44	43	44	42	43	43	47	48	47	46	47	47	48	48	77	78	78	92	94	100	88	70
P3	39	36	35	47	40	39	41	40	41	41	41	42	46	46	45	44	46	46	46	46	77	77	77	89	89	88	100	67
P1	40	36	36	49	41	40	43	42	43	42	42	42	46	47	46	45	47	47	47	48	69	68	66	68	70	70	67	100

ANI

b

	M5	M4	M2	M1	M3	M8	M7	M6	V1	Pl1	Pl2	L1	A3	A1	A2	A4	A5	A7	A8	A6	P7	P2	P6	P1	P8	P5	P4	P3
M5	100	76	74	76	0	79	77	77	0	0	0	0	0	0	0	0	0	0	0	0	0	0	0	0	0	0	0	0
M4	76	100	79	79	73	74	0	0	0	0	0	0	0	0	0	0	0	0	0	0	0	0	0	0	0	0	0	0
M2	74	79	100	91	75	0	0	0	0	0	0	0	0	0	0	0	0	0	0	0	0	0	0	0	0	0	0	0
M1	76	79	91	100	75	0	0	0	0	0	0	0	0	0	0	0	0	0	0	0	0	0	0	0	0	0	0	0
M3	0	73	75	75	100	76	0	0	0	0	0	0	0	0	0	0	0	0	0	0	0	0	0	0	0	0	0	0
M8	79	74	0	0	76	100	86	79	0	0	0	0	0	0	0	0	0	0	0	0	0	0	0	0	0	0	0	0
M7	77	0	0	0	0	86	100	83	0	0	0	0	0	0	0	0	0	0	0	0	0	0	0	0	0	0	0	0
M6	77	0	0	0	0	79	83	100	0	0	0	0	0	0	0	0	0	0	0	0	0	0	0	0	0	0	0	0
V1	0	0	0	0	0	0	0	0	100	0	0	0	0	0	0	0	0	0	0	0	0	0	0	0	0	0	0	0
Pl1	0	0	0	0	0	0	0	0	0	100	0	0	0	0	0	0	0	0	0	0	0	0	0	0	0	0	0	0
Pl2	0	0	0	0	0	0	0	0	0	0	100	0	0	0	0	0	0	0	0	0	0	0	0	0	0	0	0	0
L1	0	0	0	0	0	0	0	0	0	0	0	100	0	0	0	0	0	0	0	0	0	0	0	0	0	0	0	0
A3	0	0	0	0	0	0	0	0	0	0	0	0	100	0	0	0	0	0	0	0	0	0	0	0	0	0	0	0
A1	0	0	0	0	0	0	0	0	0	0	0	0	0	100	82	0	0	0	0	0	0	0	0	0	0	0	0	0
A2	0	0	0	0	0	0	0	0	0	0	0	0	0	82	100	0	0	0	0	0	0	0	0	0	0	0	0	0
A4	0	0	0	0	0	0	0	0	0	0	0	0	0	0	0	100	76	76	76	76	0	0	0	0	0	0	0	0
A5	0	0	0	0	0	0	0	0	0	0	0	0	0	0	0	76	100	79	79	78	0	0	0	0	0	0	0	0
A7	0	0	0	0	0	0	0	0	0	0	0	0	0	0	0	76	79	100	95	89	0	0	0	0	0	0	0	0
A8	0	0	0	0	0	0	0	0	0	0	0	0	0	0	0	76	79	95	100	89	0	0	0	0	0	0	0	0
A6	0	0	0	0	0	0	0	0	0	0	0	0	0	0	0	76	78	89	89	100	0	0	0	0	0	0	0	0
P7	0	0	0	0	0	0	0	0	0	0	0	0	0	0	0	0	0	0	0	0	100	79	79	76	80	80	79	79
P2	0	0	0	0	0	0	0	0	0	0	0	0	0	0	0	0	0	0	0	0	79	100	78	79	79	79	80	79
P6	0	0	0	0	0	0	0	0	0	0	0	0	0	0	0	0	0	0	0	0	79	78	100	76	81	80	80	81
P1	0	0	0	0	0	0	0	0	0	0	0	0	0	0	0	0	0	0	0	0	76	79	76	100	76	77	78	75
P8	0	0	0	0	0	0	0	0	0	0	0	0	0	0	0	0	0	0	0	0	80	79	81	76	100	98	92	90
P5	0	0	0	0	0	0	0	0	0	0	0	0	0	0	0	0	0	0	0	0	80	79	80	77	98	100	92	90
P4	0	0	0	0	0	0	0	0	0	0	0	0	0	0	0	0	0	0	0	0	79	80	80	78	92	92	100	88
P3	0	0	0	0	0	0	0	0	0	0	0	0	0	0	0	0	0	0	0	0	79	79	81	75	90	90	88	100
