# Supplementary material for: Verrucomicrobiota are specialist consumers of sulfated methyl pentoses during diatom blooms
Source: ISME J. 2021 Sep 7;16(3):630–41. doi: 10.1038/s41396-021-01105-7 (PMC8857213; doi:10.1038/s41396-021-01105-7)
Supplement: Supplementary file 7 — Supplementary Figure 5 [file 41396_2021_1105_MOESM7_ESM.pdf]

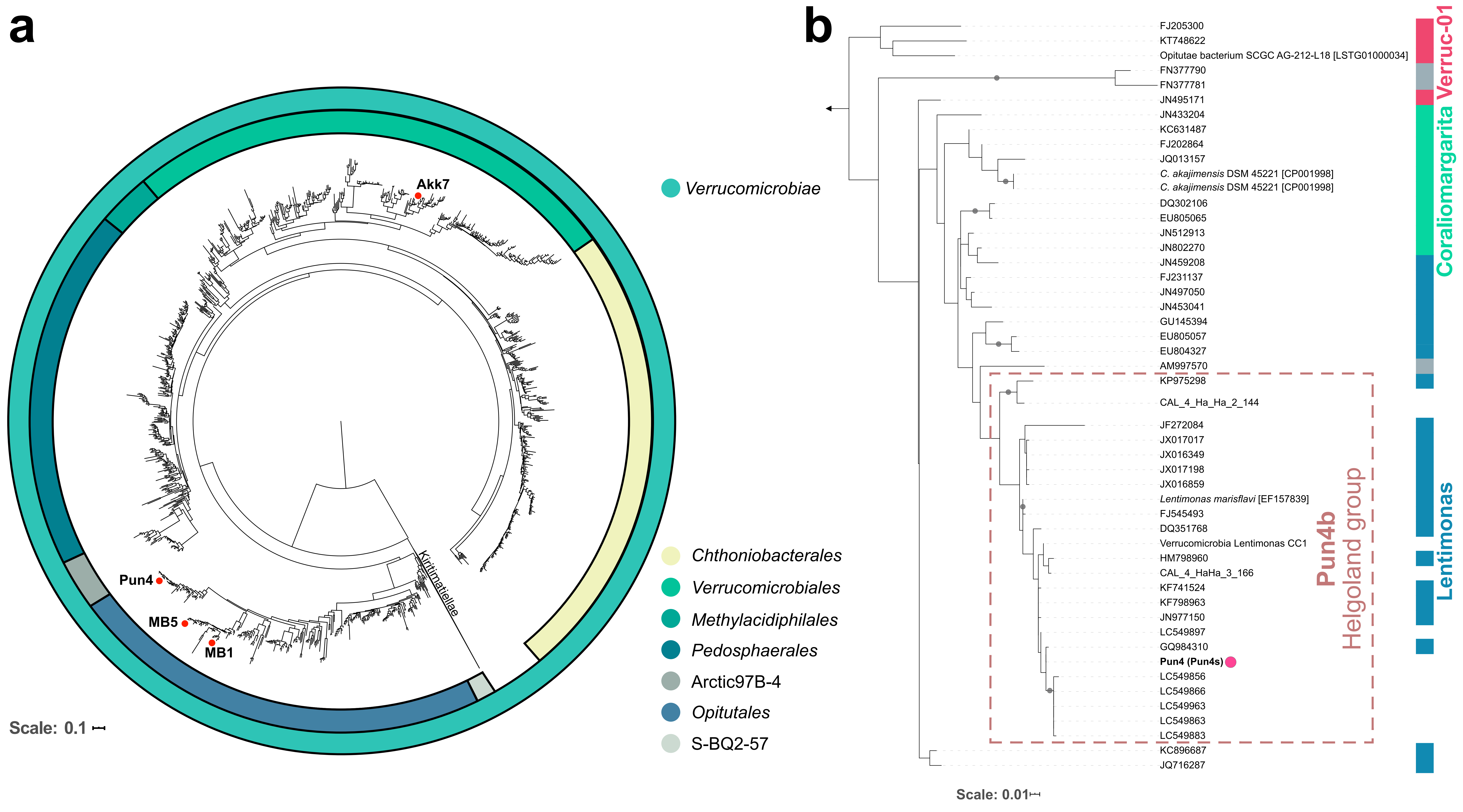

a
Akk7
Pun4
MB5
MB1
Kiritimatiellae
Scale: 0.1
Verrucomicrobiae
Chthoniobacterales
Verrucomicrobiales
Methylacidiphilales
Pedosphaerales
Arctic97B-4
Opitutales
S-BQ2-57
b
FJ205300
KT748622
Opitutae bacterium SCGC AG-212-L18 [LSTG01000034]
FN377790
FN377781
JN495171
JN433204
KC631487
FJ202864
JQ013157
C. akajimensis DSM 45221 [CP001998]
C. akajimensis DSM 45221 [CP001998]
DQ302106
EU805065
JN512913
JN802270
JN459208
FJ231137
JN497050
JN453041
GU145394
EU805057
EU804327
AM997570
KP975298
CAL_4_Ha_Ha_2_144
JF272084
JX017017
JX016349
JX017198
JX016859
Lentimonas marisflavi [EF157839]
FJ545493
DQ351768
Verrucomicrobia Lentimonas CC1
HM798960
CAL_4_HaHa_3_166
KF741524
KF798963
JN977150
LC549897
GQ984310
Pun4 (Pun4s)
LC549856
LC549866
LC549963
LC549863
LC549883
KC896687
JQ716287
Pun4b
Helgoland group
Verruc-01
Coraliomargarita
Lentimonas
Scale: 0.01
