# Supplementary figures and images for: Verrucomicrobiota are specialist consumers of sulfated methyl pentoses during diatom blooms
Source: ISME J. 2021 Sep 7;16(3):630–41. doi: 10.1038/s41396-021-01105-7 (PMC8857213; doi:10.1038/s41396-021-01105-7)

**a**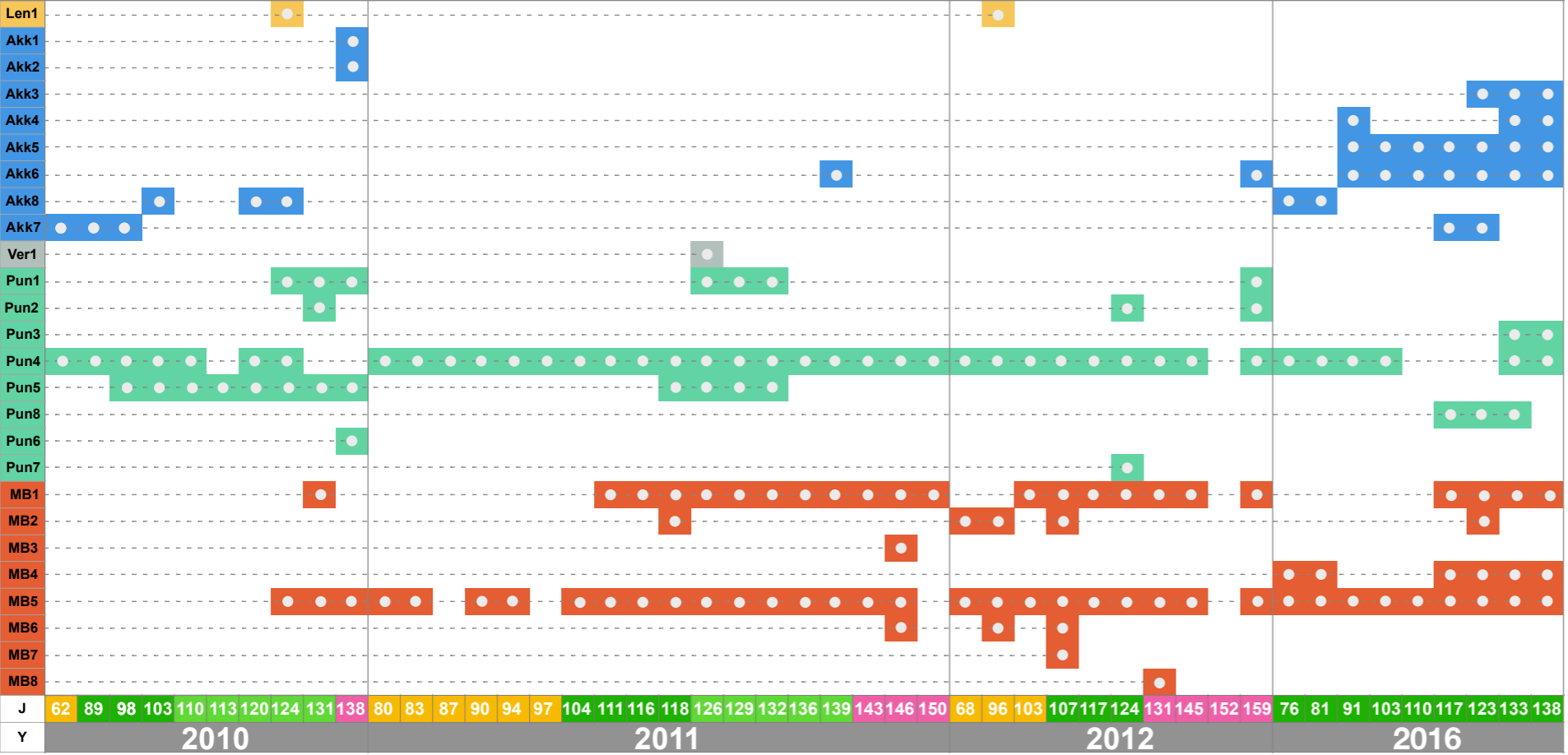**b**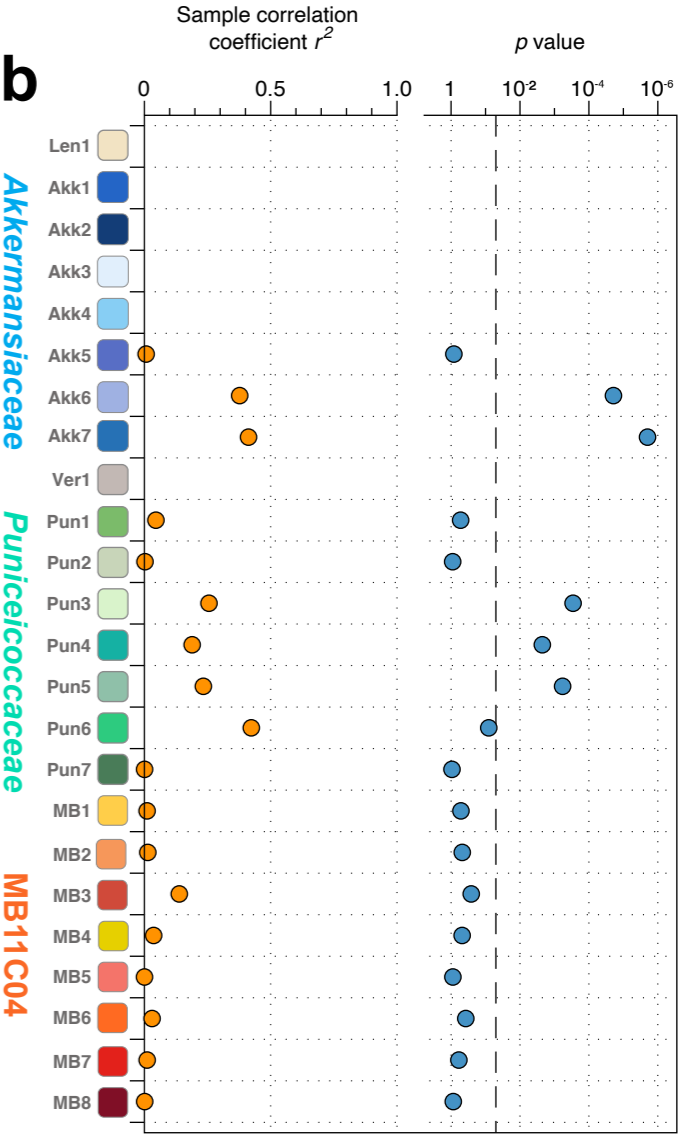

Supplement: Supplementary file 8 — Supplementary Figure 6 [file 41396_2021_1105_MOESM8_ESM.pdf]

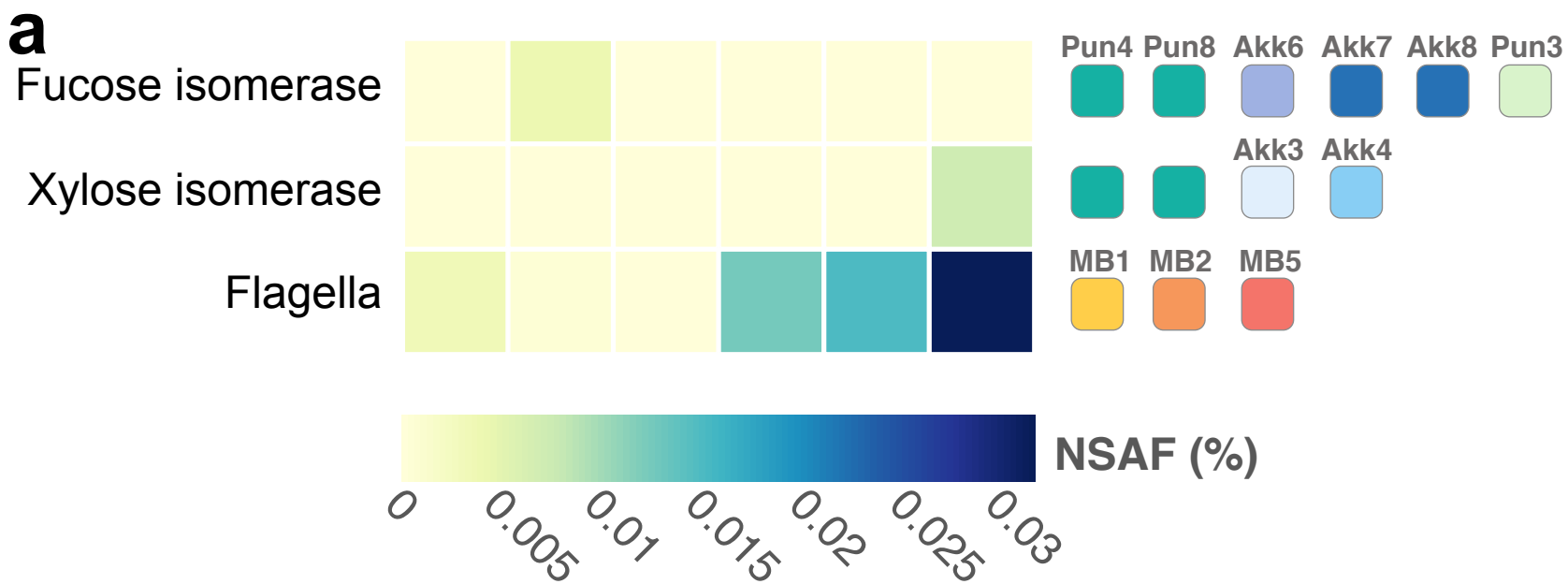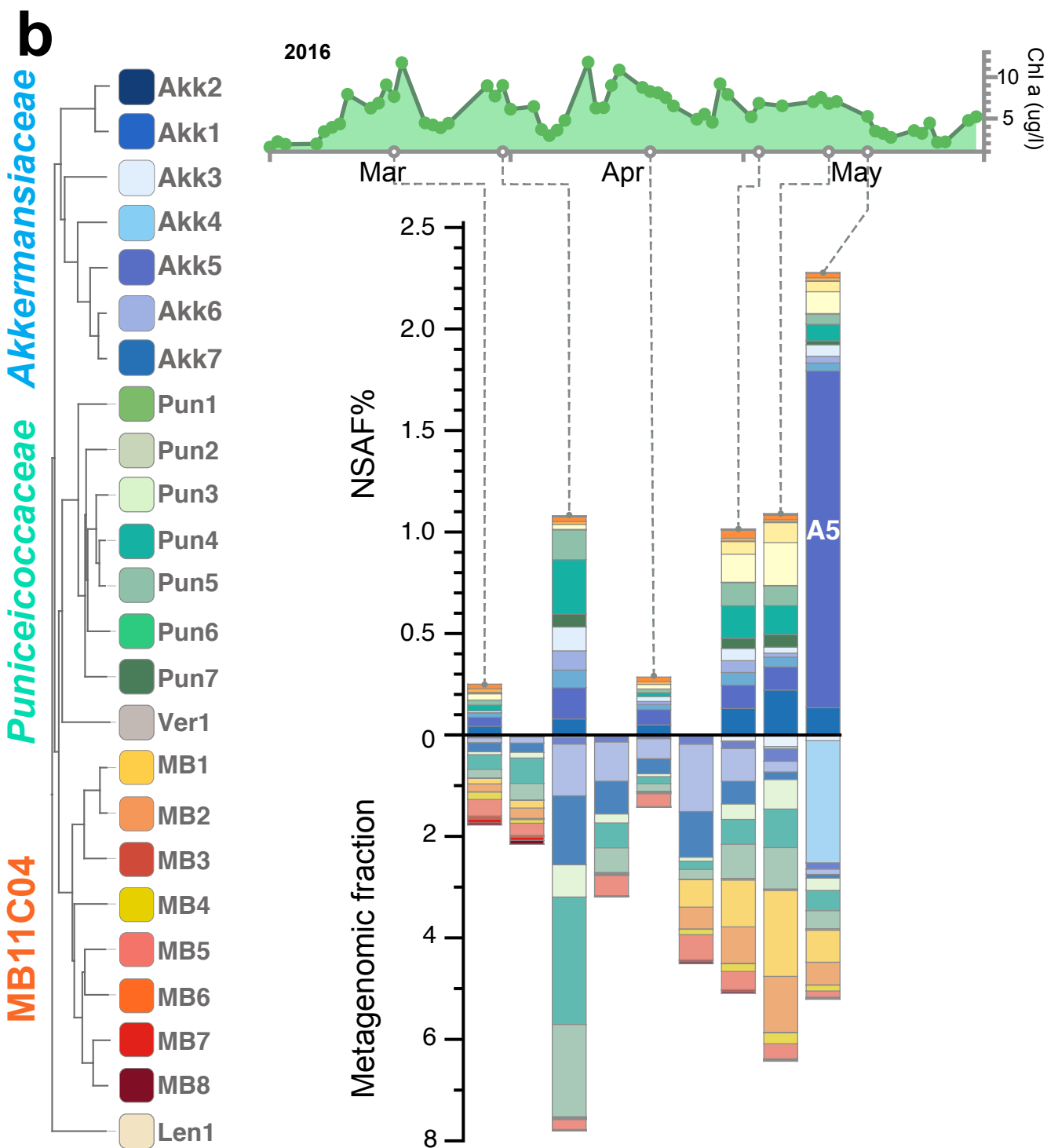

Supplement: Supplementary file 10 — Supplementary Figure 8 [file 41396_2021_1105_MOESM10_ESM.pdf]

## GH78-b

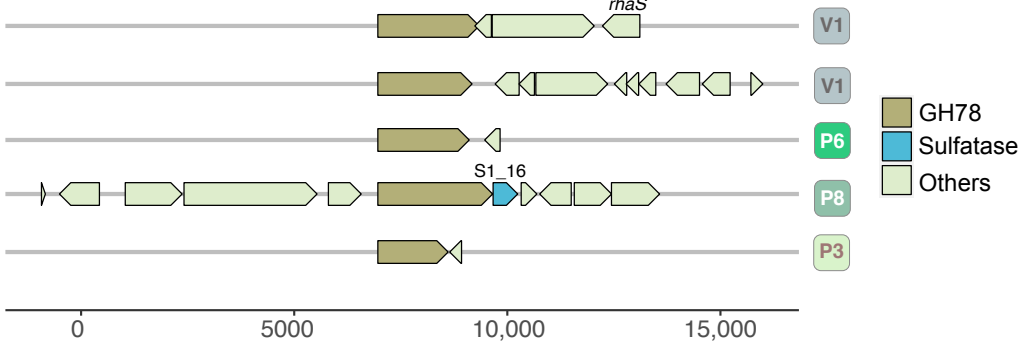

## GH78-a

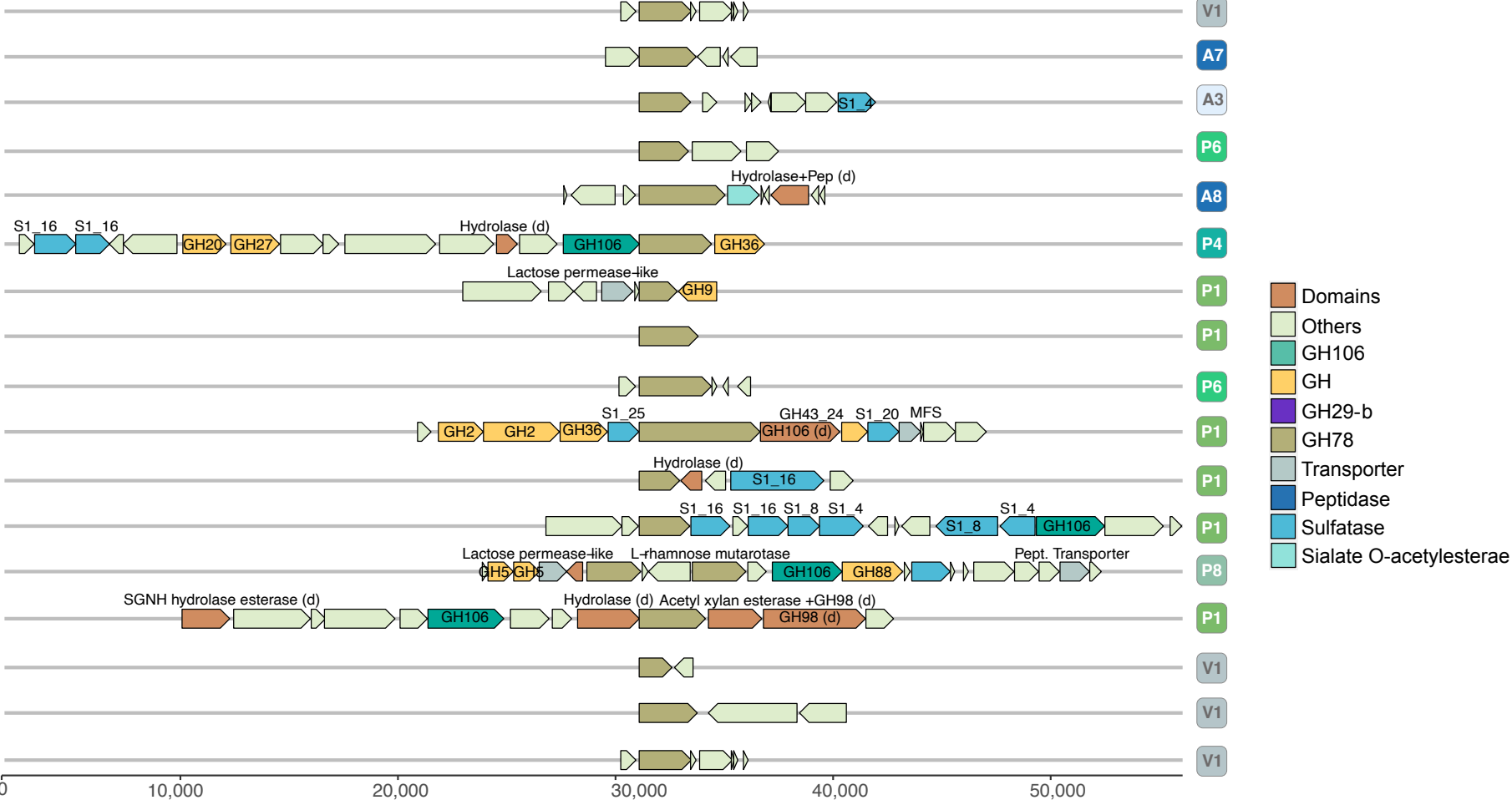

## GH106-a

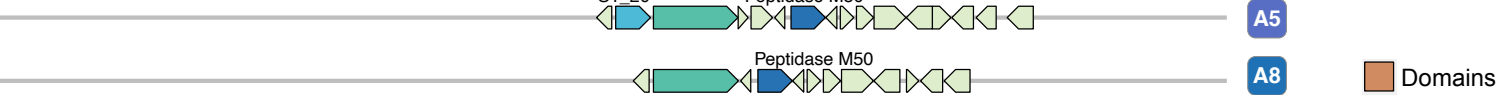

## GH106-b

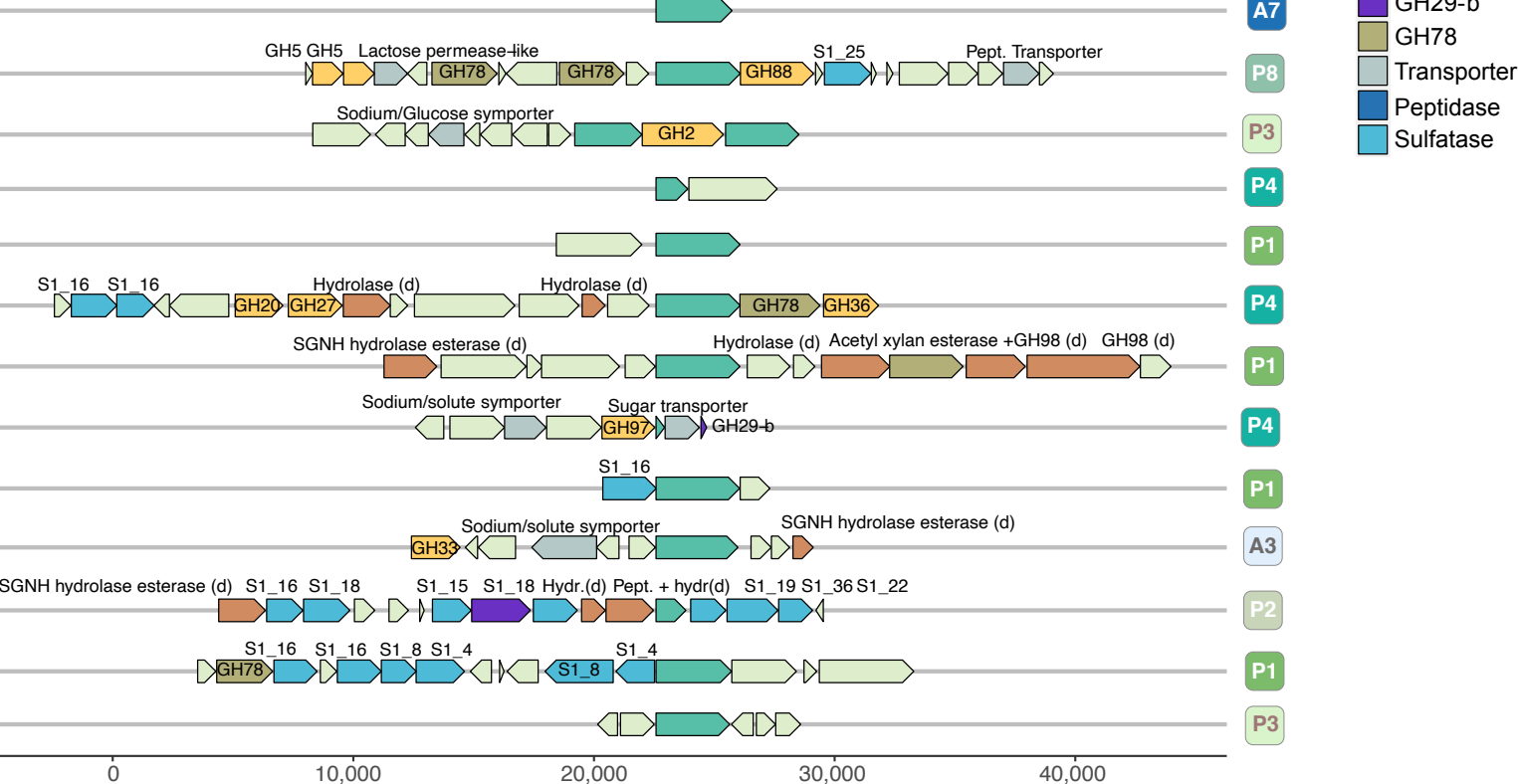

Supplement: Supplementary file 12 — Supplementary Figure 10 [file 41396_2021_1105_MOESM12_ESM.pdf]
