# Supplementary material for: Verrucomicrobiota are specialist consumers of sulfated methyl pentoses during diatom blooms
Source: ISME J. 2021 Sep 7;16(3):630–41. doi: 10.1038/s41396-021-01105-7 (PMC8857213; doi:10.1038/s41396-021-01105-7)
Supplement: Supplementary file 11 — Supplementary Figure 9 [file 41396_2021_1105_MOESM11_ESM.pdf]

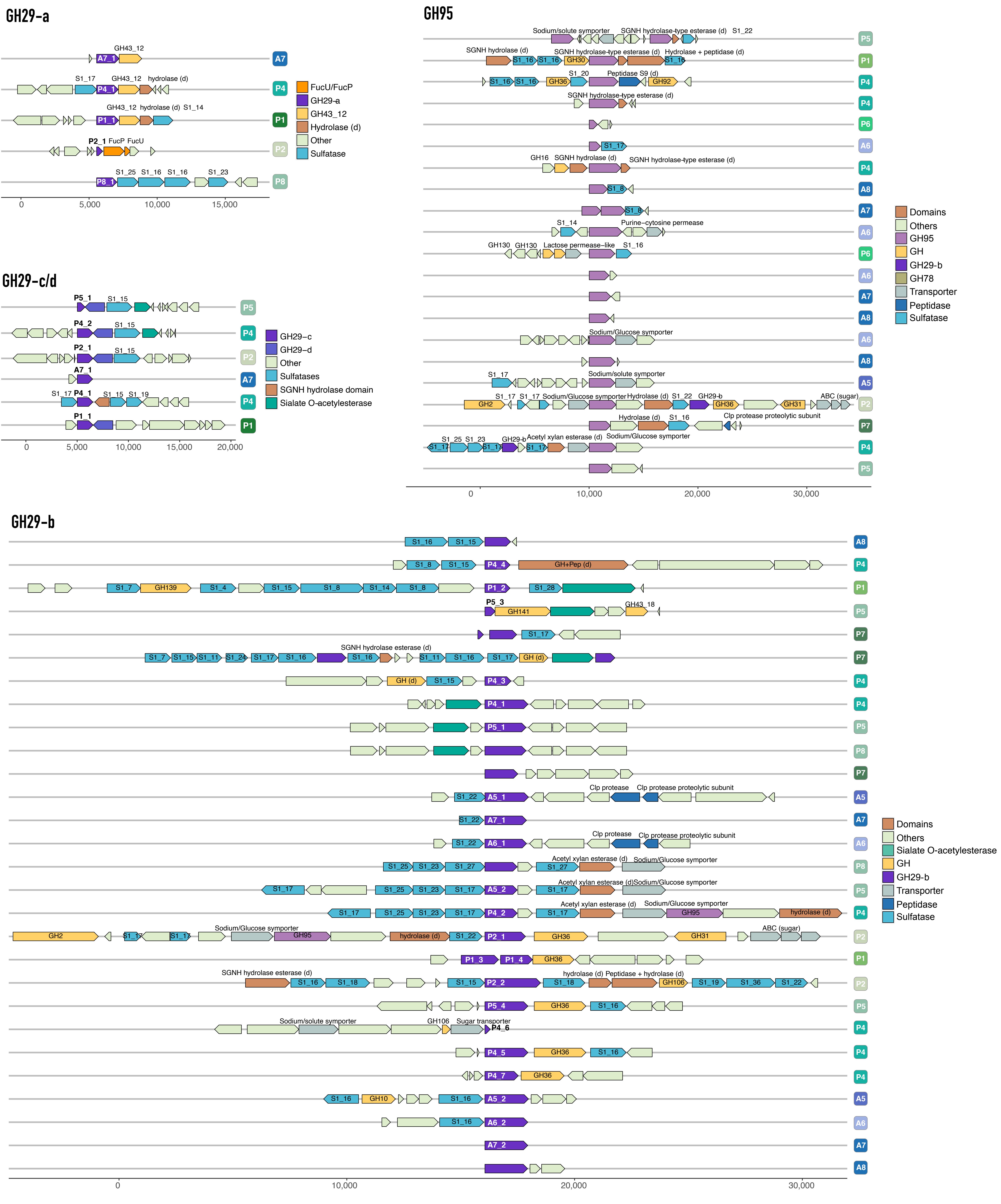

GH29-a
GH43_12
A7_1
A7
S1_17
GH43_12
hydrolase (d)
P4_1
P4
GH43_12
hydrolase (d)
S1_14
P1_1
P1
P2_1
FucP
FucU
P2
S1_25
S1_16
S1_16
S1_23
P8_1
P8
0
5,000
10,000
15,000
FucU/FucP
GH29-a
GH43_12
Hydrolase (d)
Other
Sulfatase
GH29-c/d
P5_1
S1_15
P5
P4_2
S1_15
P4
P2_1
S1_15
P2
A7_1
A7
S1_17
P4_1
S1_15
S1_19
P4
P1_1
P1
0
5,000
10,000
15,000
20,000
GH29-c
GH29-d
Other
Sulfatases
SGNH hydrolase domain
Sialate O-acetylesterase
GH95
Sodium/solute symporter
SGNH hydrolase-type esterase (d)
S1_22
P5
SGNH hydrolase (d)
SGNH hydrolase-type esterase (d)
Hydrolase + peptidase (d)
S1_16
S1_16
GH30
S1_16
P1
S1_20
Peptidase S9 (d)
S1_16
S1_16
GH36
GH92
P4
SGNH hydrolase-type esterase (d)
P4
P6
S1_17
A6
GH16
SGNH hydrolase (d)
SGNH hydrolase-type esterase (d)
P4
S1_8
A8
S1_8
A7
S1_14
Purine−cytosine permease
A6
GH130
GH130
Lactose permease-like
S1_16
P6
A6
A7
A8
Sodium/Glucose symporter
A6
A8
S1_17
Sodium/solute symporter
A5
S1_17
S1_17
Sodium/Glucose symporter
Hydrolase (d)
S1_22
GH29-b
GH2
GH36
GH31
ABC (sugar)
P2
Hydrolase (d)
S1_16
Clp protease proteolytic subunit
P7
S1_25
S1_23
GH29-b
Acetyl xylan esterase (d)
Sodium/Glucose symporter
S1_17
S1_17
S1_17
P4
P5
0
10,000
20,000
30,000
Domains
Others
GH95
GH
GH29-b
GH78
Transporter
Peptidase
Sulfatase
GH29-b
S1_16
S1_15
A8
S1_8
S1_15
P4_4
GH+Pep (d)
P4
S1_7
GH139
S1_4
S1_15
S1_8
S1_14
S1_8
P1_2
S1_28
P1
P5_3
GH141
GH43_18
P5
S1_17
P7
SGNH hydrolase esterase (d)
S1_7
S1_15
S1_11
S1_24
S1_17
S1_16
S1_16
S1_11
S1_16
S1_17
GH (d)
P7
GH (d)
S1_15
P4_3
P4
P4_1
P4
P5_1
P5
P8
P7
Clp protease
Clp protease proteolytic subunit
S1_22
A5_1
A5
S1_22
A7_1
A7
Clp protease
Clp protease proteolytic subunit
S1_22
A6_1
A6
Acetyl xylan esterase (d)
Sodium/Glucose symporter
S1_25
S1_23
S1_27
S1_27
P8
Acetyl xylan esterase (d)
Sodium/Glucose symporter
S1_17
S1_25
S1_23
S1_17
A5_2
S1_17
P5
Acetyl xylan esterase (d)
Sodium/Glucose symporter
S1_17
S1_25
S1_23
S1_17
P4_2
S1_17
GH95
hydrolase (d)
P4
Sodium/Glucose symporter
GH2
S1_17
S1_17
GH95
hydrolase (d)
S1_22
P2_1
GH36
GH31
ABC (sugar)
P2
P1_3
P1_4
GH36
P1
SGNH hydrolase esterase (d)
hydrolase (d)
Peptidase + hydrolase (d)
S1_16
S1_18
S1_15
P2_2
S1_18
GH106
S1_19
S1_36
S1_22
P2
P5_4
GH36
S1_16
P5
Sodium/solute symporter
GH106
Sugar transporter
P4_6
P4
P4_5
GH36
S1_16
P4
P4_7
GH36
P4
S1_16
GH10
S1_16
A5_2
A5
S1_16
A6_2
A6
A7_2
A7
A8
0
10,000
20,000
30,000
Domains
Others
Sialate O-acetylesterase
GH
GH29-b
Transporter
Peptidase
Sulfatase
