# Supplementary material for: Verrucomicrobiota are specialist consumers of sulfated methyl pentoses during diatom blooms
Source: ISME J. 2021 Sep 7;16(3):630–41. doi: 10.1038/s41396-021-01105-7 (PMC8857213; doi:10.1038/s41396-021-01105-7)

a

ANI95 Rep.	GTDB taxonomy					Bacterial microcompartment			Sulftases (counts)	GHs (dbCAN)				
						BMC dom	EutN/CcmL	Contiguous?		Total GHs	Fucose		Rhamnose	
	Phylum	Class	Order	Family	Genus	PF00936	PF03319		PF00884		GH29	GH95	GH78	GH106
**r198**	Bacteroidota	Rhodothermia	Rhodothermales	UBA10348	UBA10348	2	2 | 1	no	6	14	0	1	1	0
**r342**	Bacteroidota	Rhodothermia	Rhodothermales	UBA10348	UBA10348	2	3	no	11	19	1	1	1	0
**r401**	Cyanobacteria	Cyanobacteriia	Phormidesmiales	Phormidesmiaceae	Phormidesmis	2 | 3	1	yes	0	20	0	0	0	0
**r403**	Cyanobacteria	Cyanobacteriia	Phormidesmiales	Phormidesmiaceae	Phormidesmis	2 | 3	1	yes	0	27	0	0	0	0
**r219**	Cyanobacteria	Cyanobacteriia	Synechococcales	Cyanobiaceae	Synechococcus_C	2	2	no	1	7	0	0	0	0
**r180**	Proteobacteria	Alphaproteobacteria	Rhodobacterales	Rhodobacteraceae	Roseovarius	1	1	yes	1	7	0	0	0	0
**r236**	Proteobacteria	Alphaproteobacteria	Rhodobacterales	Rhodobacteraceae	HIMB11	1	1	yes	7	8	0	0	0	0
**r286**	Proteobacteria	Alphaproteobacteria	Rhodobacterales	Rhodobacteraceae	Tateyamaria	1	1	yes	3	11	0	0	0	0
**r351**	Proteobacteria	Alphaproteobacteria	Rhodobacterales	Rhodobacteraceae	UBA12010	1	1	yes	10	12	0	0	0	0
**r216**	Proteobacteria	Gammaproteobacteria	Thiomicrospirales	Thioglobaceae	Thioglobus_A	1	1	yes	0	4	0	0	0	0

b

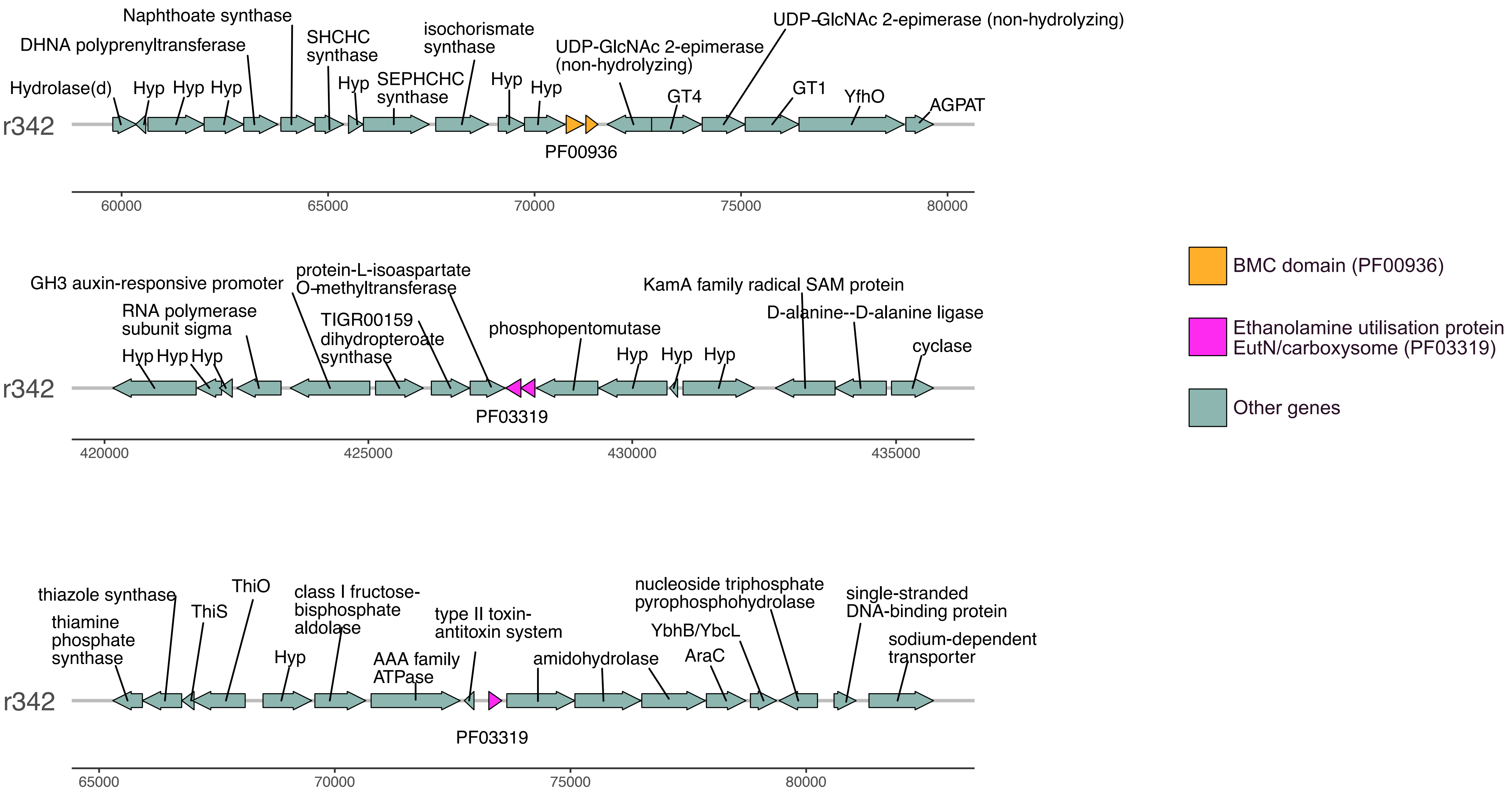

Supplement: Supplementary file 13 — Supplementary Figure 11 [file 41396_2021_1105_MOESM13_ESM.pdf]
